# Functional Enhancement and Characterization of an Electrophysiological Mapping Electrode Probe with Carbonic, Directional Macrocontacts

**DOI:** 10.3390/s23177497

**Published:** 2023-08-29

**Authors:** Radu C. Popa, Cosmin-Andrei Serban, Andrei Barborica, Ana-Maria Zagrean, Octavian Buiu, Niculae Dumbravescu, Alexandru-Catalin Paslaru, Cosmin Obreja, Cristina Pachiu, Marius Stoian, Catalin Marculescu, Antonio Radoi, Silviu Vulpe, Marian Ion

**Affiliations:** 1 National Institute for R&D in Microtechnologies–IMT Bucharest, 077190 Bucharest, Romania; octavian.buiu@imt.ro (O.B.); nicky_dumbravescu@yahoo.com (N.D.); cosmin.obreja@imt.ro (C.O.); cristina.pachiu@imt.ro (C.P.); marius.stoian@imt.ro (M.S.); catalin.marculescu@imt.ro (C.M.); antonio.radoi@imt.ro (A.R.); silviu.vulpe@imt.ro (S.V.); marian.ion@imt.ro (M.I.); 2 Termobit Prod Srl, 020281 Bucharest, Romania; cserban@fh-co.com (C.-A.S.); abarborica@fh-co.com (A.B.); 3 Fhc, Inc., Bowdoin, ME 04287, USA; 4 Faculty of Physics, University of Bucharest, 077125 Magurele, Romania; 5 Physiology and Neuroscience Department, “Carol Davila” University of Medicine and Pharmacy, 050474 Bucharest, Romania; ana-maria.zagrean@umfcd.ro (A.-M.Z.); catalin.paslaru@umfcd.ro (A.-C.P.)

**Keywords:** electrophysiological guidance electrode, directional sensing (recording) and stimulation, carbonic macrocontacts, cyclic voltammetry, electrochemical impedance spectroscopy, pulse chronopotentiometry, charge injection capacity, in vitro, in vivo

## Abstract

Electrophysiological mapping (EM) using acute electrode probes is a common procedure performed during functional neurosurgery. Due to their constructive specificities, the EM probes are lagging in innovative enhancements. This work addressed complementing a clinically employed EM probe with carbonic and circumferentially segmented macrocontacts that are operable both for neurophysiological sensing (“recording”) of local field potentials (LFP) and for test stimulation. This paper illustrates in-depth the development that is based on the direct writing of functional materials. The unconventional fabrication processes were optimized on planar geometry and then transferred to the cylindrically thin probe body. We report and discuss the constructive concept and architecture of the probe, characteristics of the electrochemical interface deduced from voltammetry and chronopotentiometry, and the results of in vitro and in vivo recording and pulse stimulation tests. Two- and three-directional macrocontacts were added on probes having shanks of 550 and 770 μm diameters and 10–23 cm lengths. The graphitic material presents a ~2.7 V wide, almost symmetric water electrolysis window, and an ultra-capacitive charge transfer. When tested with clinically relevant 150 μs biphasic current pulses, the interfacial polarization stayed safely away from the water window for pulse amplitudes up to 9 mA (135 μC/cm^2^). The in vivo experiments on adult rat models confirmed the high-quality sensing of LFPs. Additionally, the in vivo-prevailing increase in the electrode impedance and overpotential are discussed and modeled by an ionic mobility-reducing spongiform structure; this restricted diffusion model gives new applicative insight into the in vivo-uprisen stimulation overpotential.

## 1. Introduction

Deep brain stimulation (DBS) is a well-established neuromodulation therapy for the symptomatic treatment of movement disorders—such as Parkinson’s, dystonia, or essential tremor—using precisely inserted permanent (“chronic”) electrodes (“DBS leads”) into a specific brain location (the DBS “target”) and chronically delivering electrical pulses to modulate dysregulated neural circuitry [1,2]. DBS has been investigated for several other disorders: epilepsy, major depression, obsessive-compulsive disorders, Alzheimer’s disease, pain, and addiction [2,3].

The implantation of deep brain stimulation electrodes is a complex neurosurgical procedure whose overall success depends on the precision in locating relatively small and deeply located brain structures. The EM procedure—also called intraoperative neuro-monitoring—is very effective for confirming and refining magnetic resonance imaging- and atlas-based, stereotactic estimation of the DBS target [4]. During the EM, the neurophysiologist employs a dedicated computerized system to assess the anatomy along the pre-planned insertion trajectory based on microelectrode recording (MER) of extracellular single-unit action-potentials and test (macro)stimulation (STIM), using precisely driven electrode probes provided with distal, usually metallic (Pt, PtIr alloys, W), functional interfacing micro- and macrocontacts. The accurate functional mapping enabled by acute MER-STIM using these single-use probes relies on analyzing, in each explored location, the temporal patterns of neuronal firing, as well as the therapeutic effect and potential side-effects of test stimulation [5].

The most widely used EM probes for DBS are the bipolar microelectrodes, e.g., [6,7,8,9], with one recording microtip and one macro-reference contact close to each other [5]. These probes are disposable (cannot be sterilized and reimplanted due to the bioburden ensuing the insertion) and present specific constructive requirements, especially regarding the probe body (shank), which needs to be long (200–250 mm proximal–distal), ideally sub-millimeter cylindrically thin (0.5–1 mm outer diameter) and semirigid (usually metallic), as needed for deep penetrations with minimal tissue disturbances and steadily maintained directionality along the stereotactic neuronavigation path. Moreover, the probe shank may be tubular (hollow) to provide for a concentrically sliding microelectrode wire for single-unit recording, e.g., [6].

Whereas for the chronic neural probes, the scientific literature abounds with reports on novel structural and functional developments—including for those dedicated to DBS neuromodulation—almost no such references exist for the acute EM probes.

Over the last 10+ years, the reported research results that were focused on neural sensing and stimulation the long-term implants have dominantly investigated ways to enhance the electrochemical and physical properties of the functional contact material and to design new chronic electrodes and the associated technological processes [10,11,12,13,14,15,16,17,18,19,20,21,22,23]—including for DBS chronic leads. Here, the R&D has had two significant accents. One is dedicated to introducing contact site materials with optimal electrical, mechanical, and biological properties, with an emphasis on increasing the charge injection capacity for a reduced polarization overpotential and enhanced corrosion resistance [10,11,12,13,14,15,16,17,18,19,24,25,26]. Electrically conductive carbonaceous materials of various types—such as modified carbon fibers [11], glassy carbon [12,13], printed graphene [14], doped nanocrystalline diamond [16,17,27], carbon nanotubes [18,19]—have been particularly under scrutiny due to their advantageous properties: large water electrolysis window (“water window”), predominantly capacitive charge transfer, low corrosion rates, and reduced biofouling; a recent review [28] extensively covers the advantageous and problematic aspects of developing and using neural electrodes fabricated from various carbon allotropes.

The second accent is on constructive and technological solutions to develop multi-contact probes whereby the spatial distribution of the electrode contacts offers a higher geometrical selectivity to enable enhanced freedom in selecting the area to be neuromodulated and sensed according to the local somatotopic vicinity [20,21,22,23]. Exploiting the advantages offered by the constructive particularities of DBS leads—shorter, polymeric core and relatively larger diameter and lower length—the number of exposed contacts could be increased in commercial products from 4 (rings) to 12 contacts (geometrically arranged in 4 rings of 3 circumferential segments), and even 32 contacts uniformly distributed on the probe shank [20,21].

Regarding the acute EM probes, the two innovation paths mentioned above for the chronic probes are also highly needed to enable efficient, safe, and orientation-selective electrophysiological mapping. Here, the technological challenges originate from their specific constructive demands; these sum up to the need to obtain thin and long proximal-distal conductive routes on the surface of the low-diameter substrate and in addition to that, connected and precisely patterned distal functional contacts. At the same time, some of the strict requirements typical for chronic time-window interfacing electrodes—i.e., long-term chemical/electrochemical/mechanical stability and biocompatibility, as well as immunity to bioencapsulation processes—are relatively appeased in the case of the EM electrodes. The currently reported technological solutions to obtain acute multi-contact EM probes for surgical applications are based on distal exposure of metallic wires internally pulled through a polymeric probe body [29], in line with the general manufacturing method of all chronic segmented probes. Recently, an acute probe that displays directional metallic microcontacts on the sides of the electrode shaft and *circumferential* (i.e., non-directional) macrocontacts has been commercialized [30]. A similar contact architecture is offered by the macro–micro depth electrodes used for mapping and monitoring in surgical epilepsy, with platinum-fabricated, directional microcontacts and non-directional (annular) macrocontacts distributed over a polymeric shaft that can be temporarily rigidized with the help of an inner wire stylet to aid in placement [31].

We describe here the results of our recent work dedicated to functionally augmenting a commercial bipolar microelectrode probe [6] by complementing its current bipolar contact structure with additional macrocontacts (~1 mm^2^) manufactured from an electrochemically efficient carbonic material. In the lagging innovation landscape of EM probes, this development has the following goals:

1. Increasing the safety and electrical efficiency of charge injection during current stimulation using the advantageous electrochemical properties of conductive carbon;
2. Making each such macrocontact utilizable also for the recording of collectively-synchronized LFP neuronal activity, following the recent focus in functional neurosurgery research and practice [32,33,34] for improving the target identification;
3. Providing directionally-selective capabilities for the sensing and stimulation functions by patterning these macrocontacts in a circumferentially segmented geometry (offering 2–3 distinct distal-end orientations), aiming again at improving the assessment accuracy of the anatomical target and in line with the recent trends in chronic probes [22].
4. This work does not address enhancing the performance of the bipolar electrode, but its functional capabilities. In addition, the use of a specific commercial electrode for this study was made in order to include its architectural characteristics and constraints as in a realistic ‘testbed’ for this type of development.

Furthermore, the proximal–distal electrical routing is realized in a wiring-free solution, using inkjet printing or tool-based direct writing of conductive inks. The manufacturing processes were optimized in planar substrate geometry and then transferred to the body of the real probe; these were further characterized and tested in vitro using methods specific for the recording and stimulating electrodes, including voltammetry, electrochemical impedance spectroscopy, and pulse chronopotentiometry, as well in as in vivo, in adult rats. In addition, in connection with the overpotential raises observed in vivo, an unconventional in vitro experiment was conducted, whereby a simplified diffusion restriction structure was used as a modeling attempt for the underlying phenomenology.

## 2. Materials and Methods

### 2.1. Substrates

For the planar electrode mockups, glass slides of various dimensions (75 × 25 mm and 250 × 15–50 mm) were covered with self-adhesive 5 mil thick (125 μm) polyimide foils (Kapton^®^ 500HN), and the functional macrocontacts and the conductive routes were directly fabricated on the polyimide-covered surface of the commercial probes (various lengths: 10–23 cm, and outer diameters: ~550, and ~770 μm). In both cases, before patterning the functional materials, the substrate was thoroughly wiped with VLSI-grade isopropyl alcohol or ethanol, and then dried in nitrogen flow. Next, an activation treatment of the substrate surface was performed using an atmospheric plasma pen (Piezobrush PZ3 with Nearfield module, relyon plasma GmbH, Regensburg, Germany). The pen was scanned manually at uniform speed (~2 cm/s) and constant 1 mm liftoff to the treated surface, using specially designed and 3D-printed spacers. A final repeat of the alcohol wiping and nitrogen blow step was performed just before the material patterning process.

### 2.2. Functional Materials

The macrocontacts were fabricated using a commercial carbon (graphitic) paste (JELCON CH-8, Jujo Chemical Co., Tokyo, Japan) of one component polymer (synthetic polyester).

The proximal–distal electrically conductive routes were realized using various aqueous and solvent-based silver nanoparticle (Ag NP) inks with 40–50% solid content (Metalon JS-A211, and Metalon JS-A291 from Novacentrix, Austin, TX, USA; DM-SIJ-3200, and DM-SIP-3105S from Dycotec Materials, Calne, UK; Sicrys I50TM-119, and I40DM-106 from PV Nano Cell, Migdal Ha’Emek, Israel), that were processed according to producers’ specifications; all had 5B adhesion to the Kapton^®^ foil substrates and ensured as low as 1–3 Ω resistance per each longitudinal centimeter. The exterior electrical insulation—fully covering the conductive routes and only partially the carbonic contact—was realized with low-viscosity, bicomponent laminating epoxy resin (Deve Prodexim, Oradea, Romania) by hand painting with a fine-detail brush.

### 2.3. Probe Manufacturing

The printing processes were initially optimized on a planar substrate (PI-covered glass slides/plates), then transferred to the 3D (cylindrical) substrate. Both automatic and manual patterning methods were developed. As both methods provided interchangeable functional performance after their geometrical optimization, in most of the text, the patterning method specifically employed is omitted.

#### 2.3.1. Process Development on Planar Geometry Substrate

For the automatic patterning method, the long traces of Ag NP ink were deposited using an inkjet printer (Dimatix Materials Printer, DMP-2831, Fujifilm, Valhalla, NY, USA), while the distal carbonic paste patterns were written by an extrusion-based bioprinter (Allevi 1, Allevi-3D Systems, Philadelphia, PA, USA) just over and along the Ag NP lines.

For the manual patterning method, a ruling pen drawing instrument was used for both functional patterns. In this case, an Ag NP ink with higher viscosity (5–600 cPs–type DM-SIP-3105S, from Dycotec Materials Ltd., Calne, UK) than for the inkjet printing (10–12 cPs) was used, while the carbonic paste was diluted in chlorobenzene to reach the optimal viscosity for the ruling pen’s capillary retention and movement-driven flow. The dilution of the JELCON CH-8 paste was performed by employing 1,2-dichlorobenzene (DCB, anhydrous, >99%, Sigma Aldrich®, Merck KGaA, Darmstadt, Germany) as solvent. Due to its polar nature, DCB has excellent compatibility with the CH-8 paste and showed a strong dilution capability—even at low amounts of solvent, the viscosity of the paste is exponentially lowered. Optimization of the final viscosity was conducted by mixing the components in various amounts, using stirring followed by sonication (Elmasonic X-tra Basic, Elma Schmidbauer GmbH, Singen, Germany) for 30 min at 45 kHz. The optimal viscosity for the drawing process was obtained at a 1:1 paste-to-solvent weight ratio. The diluted paste was stored in a sealed vial at room temperature for further use.

#### 2.3.2. Process Transfer to the 3D Substrate

The processes optimized on the planar substrate were used with minimal differences for the microfabrication of the electrodes on the cylindrical substrate.

Applying the developed patterning processes to the cylindrical substrate asked for supplementary development of various dedicated auxiliary elements that were used for electrode alignment (in guiding channels), precise rotation (based on hollow-axis prisms), and plasma treatment liftoff (using an adapter for the plasma pen). These fixtures were fabricated using various techniques such as 3D-printing (Ultimaker BV, Utrecht, The Netherlands) using polylactic acid; laser-carving in 304 L stainless-steel plates; or dicing blade carving (Automatic Dicing Saw, DAD 322, DISCO Co., Tokyo, Japan) in 1.25 mm thick glass.

The ruling pen direct writing-based depositions were particularly straightforward for drawing longitudinal lines on cylindrical substrate geometry, as the metal jaws effortlessly glided along the ridge while naturally keeping the direction; this allowed the simple realization of multiple material paths, too.

A drying step in hot air followed each printing/writing before rotating the shaft for the next electrode printing. After the Ag NP routing was completed, they were cured in a convection oven and coated with a thin layer of epoxy resin for protection, leaving 1 cm at the distal ends exposed for the subsequent carbon material coverage and similar for the proximal end wire connections. To maximize the adhesion, the proximal ends were then plasma-pen-treated, and the segmented carbonic contacts were printed on top, allowing 1–2 mm overlaps with the Ag NP traces. Finally, the epoxy resin protection coating was carefully completed, leaving ~2 mm-long exposed carbon contacts for each electrode. For each proximal end, a thin wire was carefully aligned and connected (using conductive epoxy dots) and finally protected with epoxy resin. Most of these operations must be performed manually and supervised under magnifying lens, optical microscope, and digital microscope/endoscope camera, a common procedure for manufacturing such electrodes.

### 2.4. Characterization Methods

#### 2.4.1. Morphology and Structure

Morphology and elemental analysis of the carbonic coating were conducted using a field-emission gun scanning electron microscope (FEG-SEM) system (Nova NanoSEM 630, FEI, Williamsburg, MI, USA), and its integrated energy-dispersive X-ray spectroscopy-EDX detector (EDAX TEAM™, Pleasanton, CA, USA). The carbonic layer was also analyzed by grazing incidence X-ray diffraction (GI-XRD), using an ultrahigh-resolution X-ray diffraction system (SmartLab, Rigaku, Tokyo, Japan), and by Raman spectroscopy (alpha 300S, with Raman module, WiTec, Ulm, Germany), employing a 532 nm diode-pumped, 10 mW power laser.

#### 2.4.2. Electrochemical Characterizations

Cyclic voltammetry (CV), electrochemical impedance spectroscopy (EIS), and pulse chronopotentiometry were conducted at room temperature using an electrochemical workstation (Autolab PGSTAT302N, Metrohm, Barendrecht, The Netherlands) with Nova 1.11 software (Metrohm Autolab, Barendrecht, The Netherlands), and a conventional three-electrode electrochemical cell configuration with a 1 M Ag|AgCl reference electrode and a thick platinum wire counter-electrode. For all electrochemical analyses, a non-degassed phosphate-buffered saline (PBS) solution (10 mM PBS, 100 mM KCl) was used as the electrolyte (pH = 7.4). Slow-rate CV scans were performed at 50 mV/s, while carbon surface activation scans were performed at 100 mV/s unless otherwise specified. The voltammograms are represented as the third scan unless otherwise specified. The sweeps started at 0 V, sweeping in the positive direction first. EIS was performed with 10 mV-root-mean-square sinusoidal waveforms, with logarithmic frequency steps between [0.1 Hz, 100 kHz]. The EIS data fitting with equivalent circuits was performed with the ZsimpWin software (Echem, Lufkin, TX, USA).

For pulse chronopotentiometry, charge-balanced, symmetric biphasic, constant current pulses with zero voltage bias were generated at 1 μs sampling using a high-speed module.

Electrode stress endurance stimulation tests, in vitro recording tests, as well as the in vivo recording and stimulation experiments were performed using a clinical system for neuromodulation targeting interventions endowed with research capabilities (microTargetingTM Guideline 5, FHC Inc., Bowdoin, ME, USA). We used the system’s ability to apply current-controlled symmetric biphasic stimulation up to 10 mA, measure electrode impedance, record (up to 32 kHz sampling rate, i.e., 31.25 μs interval) the driving voltage waveforms, and calculate the half-ranges of these excursions.

For the in vitro two-electrode measurements (in PBS solution), a thick platinum wire was used as a counter-electrode, while in vivo, a stainless-steel needle electrode was inserted as a reference. The two-electrode 1 kHz impedance values are extracted automatically based on Fourier transform calculations using harmonic waveforms.

The sinusoidal signals for the in vitro recording tests were generated using a signal synthesizer with 50 Ω output impedance (HMS2525, Rohde & Schwarz GmbH & Co KG, Munich, Germany).

The material structuring for the CV and EIS characterizations was performed as follows. For the undiluted paste, 2 × 2 mm patterns were screen-printed on planar Kapton^®^ foil. For the diluted paste, the carbonic material was ruling-pen-patterned in 2 × 0.5 mm shapes on the polyimide-covered shank of the commercial probes. To act as a working electrode, both deposition types were contacted by the underneath Ag NP ink-based routes.

The activation of the cured carbonic paste was achieved electrochemically by performing a series of cyclic voltammograms (at a scan rate of 100 mV/s) in the PBS solution, either by anodization scans or by cathodic–anodic potential excursions of up to [−2, +2] V. Pulse current activation was also employed, consisting of using biphasic current pulses (120/150 μs phase duration and 150 Hz pulse frequency) of continuously increasing amplitudes (1-to-10 mA) over an interval of 30 s.

To assess the charge storage capability of the material in various conditions, the time-integral of the cathodic current in cyclic voltammograms was calculated and divided into the geometric surface area (GSA); this quantity is called in the text “specific cathodic charge storage” and abbreviated as CSC; in the case when the CV scan is conducted at slow rates (e.g., 50 mV/s) and the potential is swept over a window that is equal to the evaluated water window of the material (i.e., the voltage interval comprised between the water oxidation and reduction potentials), this leads to the so-called cathodic charge storage capacity (CSC_C_) [25]. This is one of the parameters that is used to qualify the charging capability for stimulating electrodes—albeit rather for benchmarking purposes due to drastic differences—in signal dynamics and corresponding phenomenology—between the slow-sweep voltammetry and pulsed current flow.

### 2.5. In Vivo Recording and Stimulation

Experiments were performed in two adult male Wistar rats, fully anesthetized with a solution of chloral hydrate (5%, 1 mL/kg) administered intraperitoneally (i.p.). During the procedure, the depth of anesthesia was monitored by assessing the absence of hind limb withdrawal reflex and was maintained by additional i.p. doses of anesthetic (1/4 of the first dose). The anesthetized animals were placed in a stereotaxic frame (RWD Life Sciences, Shenzen, China) and provided with ear and bite bars. The surgical procedure involved the exposure of the skull by removing the skin and the periosteum 3 mm anterior, 5 mm posterior, and 5 mm lateral on both sides from bregma suture, and the drilling of bilateral circular craniotomies of 2 mm diameter, 2.4 mm posterior, and 2 mm lateral to bregma on both sides.

## 3. Results

### 3.1. Constructive Concept

**Figure 1:**
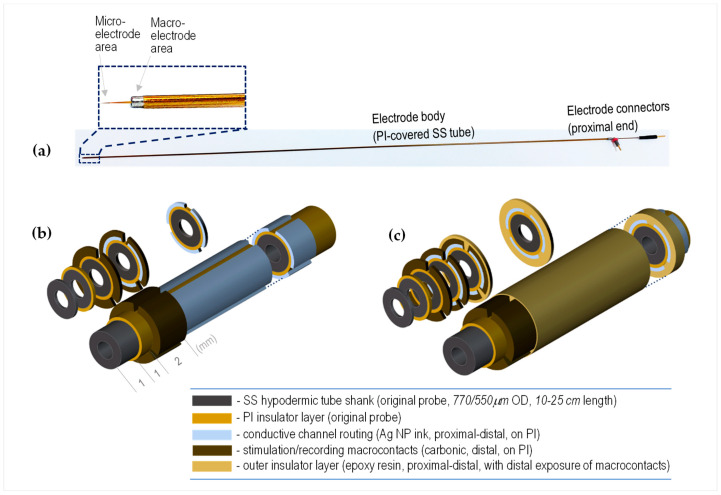
(**a**) Image of a clinical microelectrode probe (type 34685L/Z, FHC, Inc., Bowdoin, ME, USA), with distal-end detail of the stainless-steel macrocontact and tungsten (or platinum-iridium) wire microcontact; (**b**,**c**) constructive concept of the novel EM probe with complementary carbonic macrocontacts on the surface of the original microelectrode. General view and axial cross-sections: (**b**) without the outer insulation, and (**c**) completed with the outer insulation.

The original bipolar microelectrode probe [6] (Figure 1a) incorporates a polyimide (PI)-insulated tungsten, or Pt-Ir, fine-sharpened wire with a very small active exposed tip zone (<50 µm long) to create a microelectrode contact that is used mainly for the sensing of extracellular single-unit action-potentials. The microelectrode wire is concentrically sliding within the stainless-steel (SS) tubular shank of the probe, such that a specified length of the former (e.g., 10 mm) can be pushed out into the neural tissue to allow MER measurements. The SS shank is PI-insulated throughout, presenting a circumferential exposure at the distal end to form a circular macrocontact of ~1 mm in the axial direction. The macrocontact is used as an electrical reference during MER and as a macrostimulation electrode (paired with an insertion guiding tube). At the proximal end of the probe, pin connectors are made available to allow cable connections to the microelectrode wire and the macrocontact.

The present work aimed at adding segmented distal carbonic macrocontacts, equally located angularly around the distal PI cover of the SS shank of the probe. As depicted in Figure 1b,c, the constructive concept resides in adding these new functional channels directly on the PI insulation of the original probe. This is realized by printing/writing both the electrically conductive proximal-to-distal channel routes—using Ag NP ink—and, partially overlapped in the axial direction with the former, the circumferentially segmented carbonic contacts—using the carbonic paste. As the outer diameter (including the 60–70 μm thick PI layer) of the original probe was in the range of 550 μm, or 770 μm for a thicker version, the choice at this time was to realize up to 2 opposed macrocontacts (angular 180°) on the thinner type, and up to 3 macrocontacts (angular 120°) on the thicker one. In the axial direction, the added carbonic macrocontacts are extended starting with 1 mm proximal from the distal end of the PI insulator sleeve, with an exposed length of ~2 mm.

In this development, particular challenges in the realization of functional material patterning are associated with:

- The non-planar shape of the substrate, characterized by a small radius of curvature (~280–490 μm), as well as the high aspect ratio (up to ~600, for the 23 cm long electrodes);
- The need to make the depositions over an electrically insulating layer, a fact that limits the temperature regime of the subsequent processes;
- The need to realize the patterns as separated (segmented) parallel lines along several generators of the cylindrical shaft;
- In addition to the need to rotate the shaft for each such channel, each material needs a post-curing treatment to become functional.

Various techniques and methods were tested to solve these problems, including those based on classical microtechnology processes; the best solutions converged to using paste and ink-based materials and two classes of methods: automatic—using inkjet printing of Ag NP inks for the long proximal–distal routes, and extrusion printing for the carbonic paste patterns; and manual—using a ruling pen, an approach that proved to be particularly convenient and efficient for the uniform drawing of low-viscosity materials along a cylindrical surface.

### 3.2. Characterization of the Carbonic Material

#### 3.2.1. Morpho-Structural Analysis

The carbonic paste, ultimately driving the electrode–tissue interface functionality, was first characterized in-depth, by SEM, EDX, Raman, XRD, and electrochemically (CV, EIS), to assess its benefits for the application.

**Figure 2:**
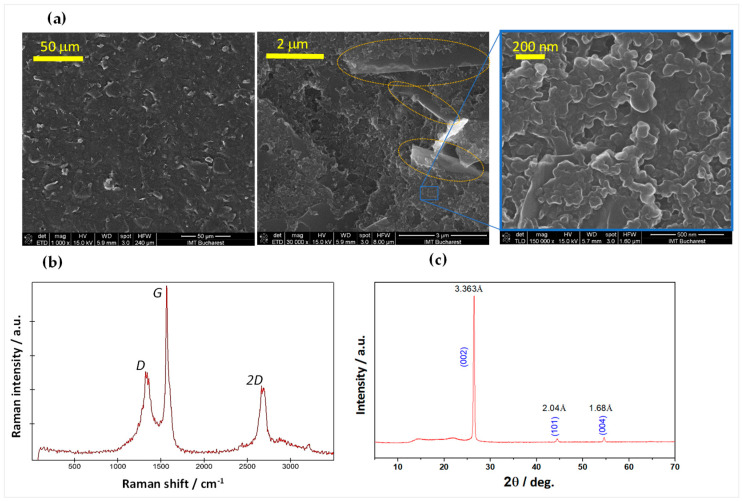
(**a**) Multiscale SEM images of the thermally cured carbonic paste. In the 30,000 × magnification (center), few-micron-sized lamellar graphitic insertions are distinguished (dotted ovals). (**b**) Raman spectrum of the cured carbonic material deposited on polyimide (Kapton^®^ foil). (**c**) XRD pattern of the cured carbonic material deposited on polyimide (Kapton^®^ foil). See Supplementary Materials for other details on the material and measurements.

SEM and EDX characterization of the carbonic layer (Figure 2a and Figure S1) at 1000×, 30,000×, and 150,000× magnifications reveal a porous structure that incorporates distinctive graphitic lamellar flakes of a few-micron size, with a content of 92–94% carbon and 6–8% oxygen (Figure S1). Although a quantitative characterization of the specific surface area and pore structure was not in the scope of this study, this morphology signals a relatively large electrochemical area that is generally beneficial for lowering the electrode impedance and increasing the charge injection.

Raman spectroscopy analysis (Figure 2b) reveals the three dominant bands caused by resonant phonon vibrations of graphitic materials: D, at 1332–1338 cm^−1^; G, at 1566–1569 cm^−1^; and the 2D, or G′, overtone at la 2687–2694 cm^−1^ (Figure S2). The relatively low intensity of the defect-induced D band to the G band’s intensity, the relatively large intensity of the G′ overtone, as well as the low full width at half-maximum (FWHM) of the G band (~35 cm^−1^, when fitted with a Lorentzian function), are factors that indicate a good-quality sp^2^ carbon hybridization content.

The graphitic quality of the carbonic material was also confirmed by the GI-XRD characterization (Figure 2c), where a major diffraction peak is observed at 26.5° corresponding to the (0,0,2) graphitic plane, indicating a d_002_ = 3.36 Å interplanar distance by Bragg’s law. This pattern strongly denotes a high structural order close to hexagonal graphene (2H-polytype) with a graphitization degree of ~89% [35].

In view of graphite’s well-known biocompatibility, chemical stability, large water window, and electrical conductivity, these SEM, EDX, Raman, and XRD analyses, as well as the cured paste’s excellent abrasion resistance and adhesion to the PI substrate and to cured Ag NP inks, are all prefiguring a material with good potential for the targeted role in this application.

The Ag NP inks used in this work showed good sintering properties after the curing treatment at the manufacturers’ specified temperatures, as well as very good adhesion on polyimide and in the carbonic paste stacking (Figure S3).

#### 3.2.2. Cyclic Voltammetry and Electrochemical Impedance Spectroscopy

Extensive electrochemistry assessments were performed for various scan ranges, GSAs, and electrochemical activation methods, for both the undiluted and diluted forms of the carbonic material. As the overall CV and EIS measurements (performed on various electrode configurations) presented good interelectrode uniformity, as well as a very good functional equivalence between the undiluted and diluted forms of the material, we present here some of the most relevant results to offer various views of the material performance.

**Figure 3:**
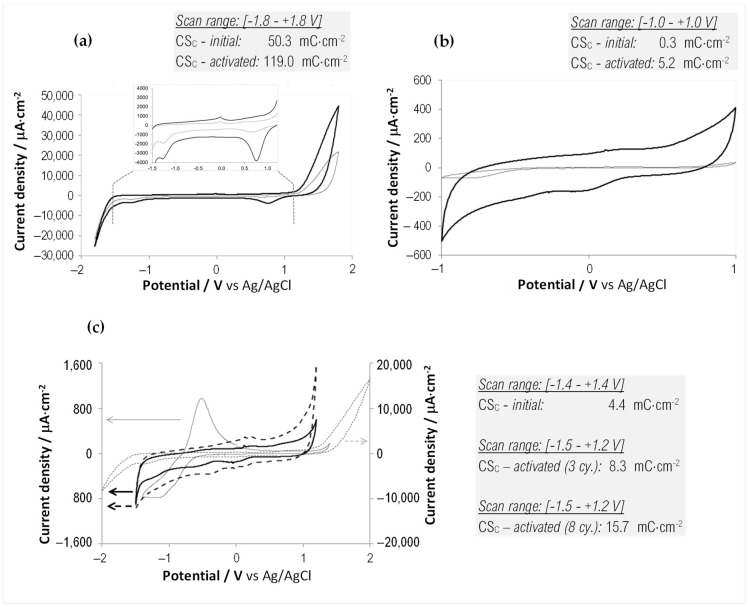
Representative CV scans and calculated specific cathodic charge storage CS_C_ for the *undiluted* (**a**,**b**) and *diluted* (**c**) carbonic paste before and after electrochemical activation. (**a**) CVs between [−1.8, +1.8] V were recorded before (*gray trace*) and after anodic activation (*black trace*). GSA: 4 mm^2^. Scan inset: scan detail in the [−1.5, +1.2] V range. Inset box: CS_C_ before and after 3 anodic activation cycles ([0, +1.8] V @ 100 mV/s). (**b**) CVs between [−1.0, +1.0] V were recorded before (*gray trace*), and after anodic activation (*black trace*). GSA: 4 mm^2^. Inset box: CS_C_ before and after 3 anodic activation cycles ([0, +1.8] V @ 100 mV/s). (**c**) Results for the diluted paste. GSA: 1 mm^2^. CV before activation, range [−1.5, +1.2] V (*continuous gray trace*). CV (at 100 mV/s) during the 3rd symmetric activation cycle, range [2, +2] V (*dotted gray trace*, right ordinate axis). CV after 3 symmetric activation cycles, range [−1.5, +1.2] V (*continuous black trace*). CV after 8 activation cycles, range [−1.5, +1.2] V (*dashed black trace*). Inset box: CS_C_ before and after 3, and 8, symmetric activation cycles ([−2, +2] V @ 100 mV/s).

For the undiluted carbonic material, cyclic voltammetry slow-rate scans (Figure 3a,b) revealed that, at a conservative estimation, the water window lies approximately between −1.5 and +1.2 V. The scans are almost identical to that reported for graphene-ink-printed electrodes reported in [14]. As shown in the inset boxes, the specific cathodic charge storage presents a significant increase following an anodic electrochemical activation (three CV cycles in the [0, +1.8] V range at 100 mV/s scan rate). As mentioned in the Section 2, throughout this work, it was chosen to generalize the usual “cathodic charge storage capacity” (CSC_C_) rating parameter with the more flexible CS_C_ to provide the sometimes-needed comparative insight on the changes in the stored charge induced at various potential windows and scan rates. Therefore, CS_C_ is calculated in some cases by discarding the slow-rate and water window-range constraints. At the same time, the actual CSC_C_ can be learned when these conditions are satisfied (such as in Figure 3c and Figure S8).

For the diluted paste, CV results are presented in Figure 3c. Following a symmetric electrochemical activation between −2 and +2 V, it is noticed that the initial reversible anodic peak at −0.5 V vanishes.

As shown in the figure and the box inset, the number of activation cycles significantly affects the charge storage parameter. As shown later in the text, this symmetric potential activation is stable even after a long time of storage in the open air and aggressive current pulse tests.

The absence of redox features in the voltammograms of the activated carbonic paste corroborates with its graphitic nature—revealed by the XRD and Raman analyses—and denotes that the material will transfer the charge massively by capacitive displacement current. The absence of faradaic reactions is an asset for stimulating electrodes, as it shows that no chemical species are generated or used up to sustain the charge transfer, therefore ensuring both higher procedural safety and chemo-structural stability of the electrode [24,25,26].

**Figure 4:**
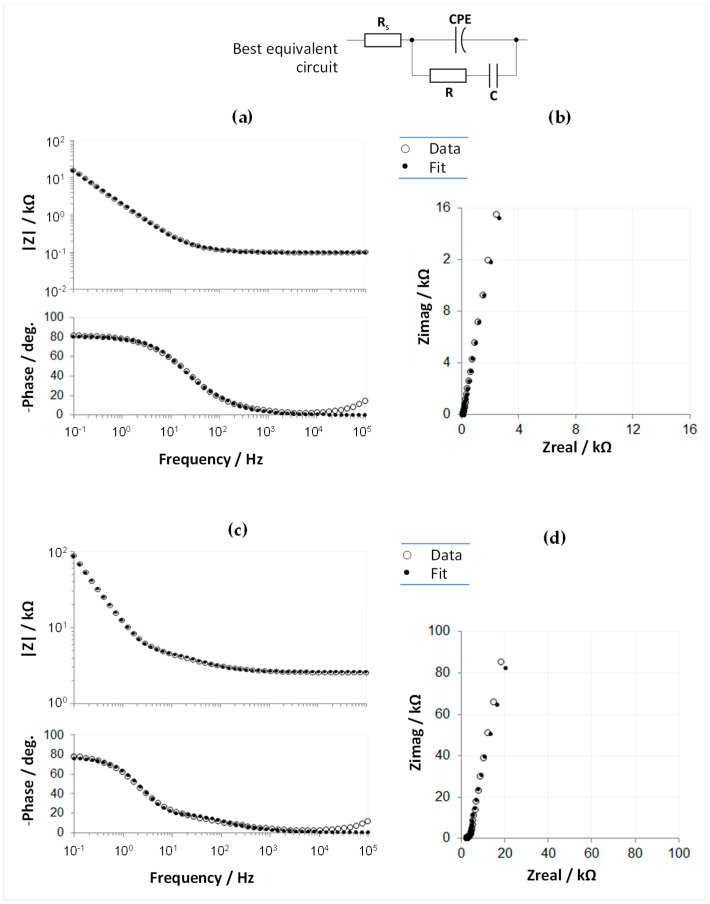
Representative EIS results for the *undiluted* (**a**,**b**), GSA: 4 mm^2^—and *diluted* (**c**,**d**), GSA: 1 mm^2^—electrochemically-activated carbonic paste. Measured Bode diagrams (**a**,**c**), and Nyquist plots (**b**,**d**), superposed with the corresponding calculated fits. The fits were based on the best equivalent circuit presented in the figure top. See Supplementary Materials for method details and an illustrative EIS data comparison before and after carbon surface activation (Figure S4).

EIS data were collected on the same probes to characterize the nature of the electrode-electrolyte interface, and the equivalent circuit model was extracted for the undiluted and diluted forms of the carbonic material (Figure 4).

After informed considerations and extensive trials, the model that optimally and robustly described the collected data was found to be identical for the two forms of the carbon paste, consisting of the purely resistive general contribution mainly due to the electrolyte (R_S_) and two branches in parallel: a constant phase element (CPE), and an R-C series group. This is a slightly modified version of a model that describes graphitic electrodes that incorporate superficial micro-orientation structures with non-basal plane exposures [36], as substantiated by the SEM observations (Figure 2). The fact that the double-layer capacitance branch required modeling by a CPE element agrees with the SEM-observed roughness and porosity of this material, as this morphology introduces a non-ideal capacitive behavior, also known as frequency dispersion [37]. More explanations on the involved CPE element and the best-fitting values extracted for the equivalent circuit elements are given in the Supplementary Materials and Supplementary Table S1.

The overall characterization results concluded that the undiluted and diluted forms of the graphitic paste present only minor and nonsignificant functional differences and can be used interchangeably for the direct patterning of the electrode macrocontacts.

Given the unknown/uncontrollable conditions occurring in vivo, a non-degassed PBS solution was employed during these experiments. Whereas bubble formation or irreversibility was observed, the oxygen content may interfere with the actual values of the water window, CSc, and EIS analysis; therefore, the reported quantities might present slight variations in an oxygen-depleted environment.

### 3.3. Planar Substrate Probes

Process optimization for realizing the Ag NP ink traces and the carbonic contacts was performed first in the planar substrate geometry. The electrodes realized in planar geometry were designed as “functional mirrors” of the targeted (i.e., cylindrically patterned) electrodes. Accordingly, their fabrication methods, underlying material (i.e., Kapton^®^), geometrical parameters, and spatial resolutions were identical. Hence, importantly, besides being a convenient breadboard for process optimization, the planar version also becomes a functional equivalent of the real cylindrical electrode, enabling cheap and convenient interchangeability for subsequent functional assessments.

An in-depth presentation of the planar substrate probe fabrication and constructive details, as well as their various characterization steps, are presented in the Supplementary Materials. The extensive studies presented—related to: the sweep frequency effects, the endurance to various realistic electrical stimulation levels, the impact of various electrochemical activation strategies, and the quality of in vitro signal sensing using a floating voltage configuration [38] — enabled helpful insight into the probe operation and capabilities, and supported the constructive concept for further cylindrical geometry development.

### 3.4. Cylindrical Substrate Probes

**Figure 5:**
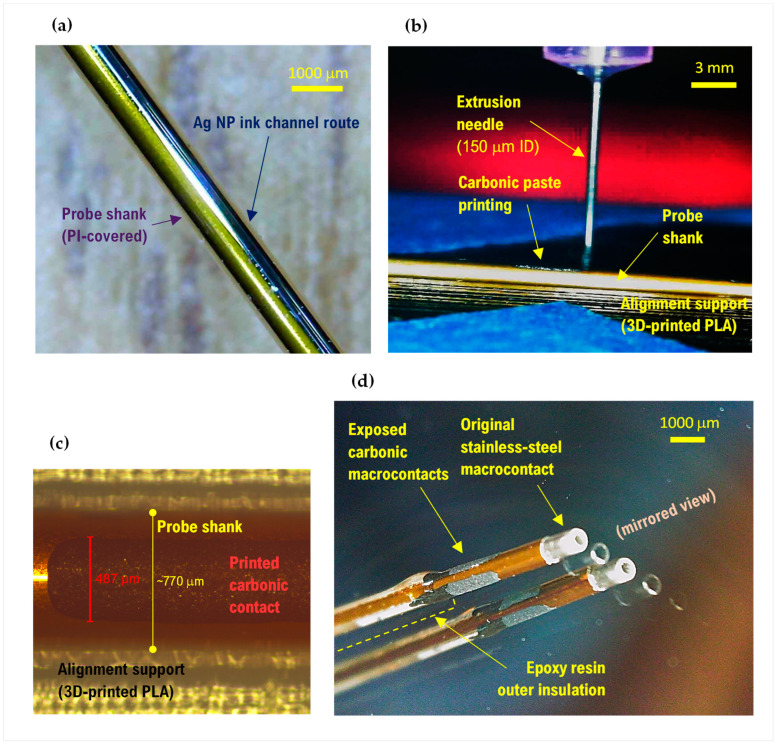
Illustrative optical and optical microscopy images for the manufacturing of cylindrical geometry probes. (**a**) Proximal–distal conductive route. (**b**) Timeframe during extrusion printing of a carbonic macrocontact. (**c**) Microscopy evaluation of the macrocontact. (**d**) Distal-end detail of a 3-macrocontact probe (angular 120°) on a 770 μm outer diameter, 23 cm long probe shank; mirror-view for better circumferential examination.

The processes developed in the planar substrate geometry stage were transferred to the bipolar microelectrode, as described in the Section 2. The probe samples realized on 550 and 770 μm diameter shanks of 10 to 23 cm length comprised 2 and 3 longitudinal electrode channels, with the distal graphitic macrocontacts of ~1 mm^2^ GSA being circumferentially segmented towards 2 and 3 radial views (180° or 120° orientations, respectively). Figure 5 displays relevant phases of the development cycle and a detailed distal-end view of a 3-macrocontact probe.

#### 3.4.1. Electrochemical Assessments

**Figure 6:**
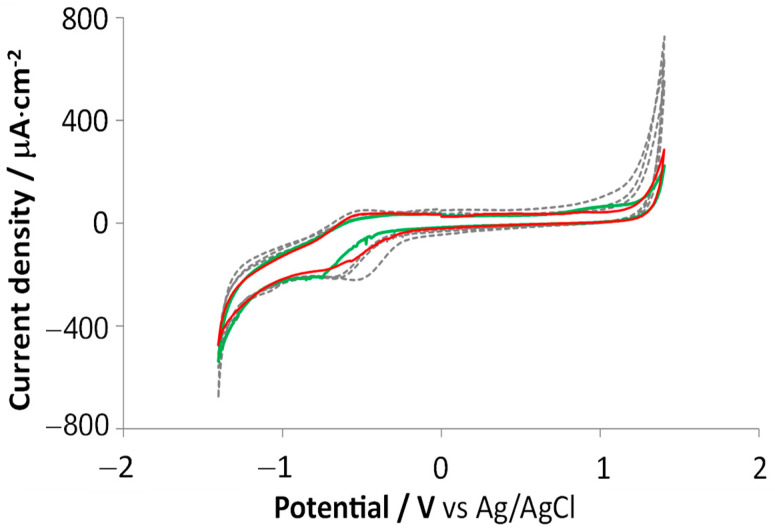
Comparative CV scans for the five electrodes in both substrate geometries. Scan interval: [−1.4, +1.4] V. Small oxygen reduction peaks are observed in the (−0.8, −0.5) V range. Planar electrodes P1–P3—dashed traces; cylindrical electrodes Ca, Cb—continuous traces. Scan rate: 50 mV/s. GSA: 1 mm^2^.

Representative results of a comparative CV and EIS study performed in PBS solution on three planar substrate electrodes (P1–P3) and two cylindrical substrate electrodes (Ca, Cb) are presented in Figure 6 and Table 1, respectively. Electrodes Ca and Cb are printed on a 770 μm diameter shank at 180° oriented views. All five electrodes have carbonic contacts automatically printed by extrusion. For channels P1–P3, the electrochemistry analysis was performed following a stimulation stress test, while channels Ca and Cb were analyzed just after a pulsed current activation session. These data show that the process transfer from planar to cylindrical geometry preserves well—to the limits of process precision—the electrochemical performance of the electrodes so that both electrode types can be used interchangeably for functional characterizations.

#### 3.4.2. Charge Injection Capacity

In vitro evaluation of the charge injection capacity was conducted using 3-electrode high sampling rate (1 μs) pulse chronopotentiometry in PBS. The charge injection capacity (CIC, or Q_inj_), sometimes referred to also as charge injection limit, is an essential qualifying measure for the stimulation function of the electrodes, as it indicates the maximum charge per unit area that can be safely injected through a stimulating electrode without reaching neither the H_2_ evolution in the cathodic phase nor the O_2_ evolution in the anodic phase of the stimulation waveform [25,39,40].

For this analysis, current-controlled, biphasic (with cathodic leading phase), charge-balanced pulse waveforms were employed, with a 30 μs interphase gap to better assess the driving voltage excursion stages. The current amplitudes were tested to up to 9 mA, while the phase duration and the pulse train frequency were chosen to 150 μs, and 150 Hz, respectively; these values represent realistic, upper-limit parameters that can be encountered in an EM protocol, while in some combinations they exceed the Shannon charge density limit [41,42], that was established mainly for chronic stimulation. As in this work, the aim is to assess the technical merits of the proposed materials and design, while the focus is on testing their technical margins when subjected to fast-pulse stimulation waveforms relevant to the EM procedure. For further details on this experiment, see also the Supplementary Materials.

**Figure 7:**
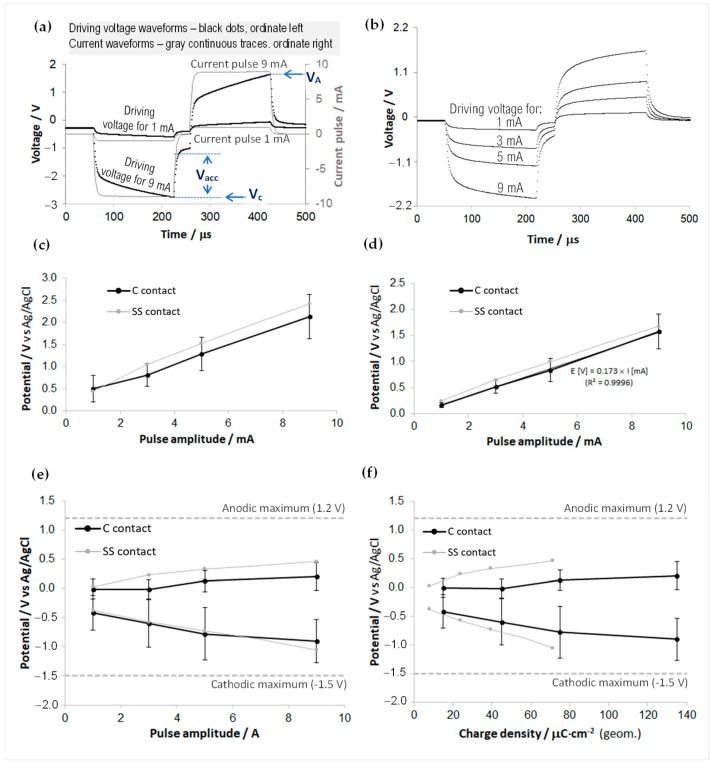
Pulse chronopotentiometry analysis for 1 mm^2^ GSA carbonic contacts. (**a**) Example of 1 and 9 mA amplitude, biphasic current pulses superposed with representative driving voltage waveforms. (**b**) Representative driving voltage waveforms for 1, 3, 5, and 9 mA current pulse amplitudes were recorded in the chronopotentiometry experiment for CIC evaluation. (**c**,**d**): Cumulative results from the pulse chronopotentiometry measurements for 1 mm^2^ GSA carbon-contact electrodes (*n* = 5), and one 2 mm^2^ GSA stainless-steel (SS) contact. The error bars correspond to the standard deviation (one measurement per each carbonic channel measured). (**c**) Driving voltage half-ranges (*V_A_* − *V_C_)/2*) vs. the current pulse amplitude. (**d**) The access voltage (V_acc_), evaluated from the voltage waveforms vs. the current pulse amplitude; the linear fit for the carbonic contact indicates an estimated access resistance of 173 Ω. (**e**,**f**): Cumulative results for the interfacial polarization extremes (E_C_ and E_A_), calculated from the pulse chronopotentiometry measurements (150 Hz biphasic pulses, 150 μs phase duration), after corrections for V_acc_, for the 1 mm^2^ GSA carbon-contact electrodes (*n* = 5), and one 2 mm^2^ GSA stainless-steel (SS) contact. The overlayed −1.5 V cathodic and +1.2 V anodic maxima were estimated from CV scans at 50 mV/s, for the carbon-contact electrodes. The error bars correspond to the standard deviation (one measurement per each carbonic channel). (**e**) Plots vs. the current pulse amplitude. (**f**) Plots vs. the charge density per electrode GSA. See Supplementary Materials for additional details.

Figure 7 presents the measurements and the synthetical results of this analysis. Figure 7a,b display typical current pulse waveforms and driving voltage excursions (between *V_C_* and *V_A_*) recorded during the experiments. The waveform of the voltage response reflects the polarizable nature of the electrode and is a complex result of various polarization mechanisms [25,40,43].

The rising character of the voltage waveforms during the constant current phases of the pulse reflects the capacitive nature of the electrode-electrolyte interface, and the extents of these transients in the cathodic and anodic phases are the targets of the CIC estimation [17,25,39,40]. To obtain these cathodic to anodic interfacial excursions (*E_C_* = *V_C_ + Vacc* to *E_A_* = *V_A_ − Vacc*), the voltage waveform must be corrected for by extracting the so-called *access voltage,* or *“iR drop”* (*V_acc_*), that is, the purely ohmic contribution to the overpotential waveform and is recognizable in fast sampling rate recordings as the abrupt voltage dips/jumps occurring at the edges of the pulse phases.

The analysis was conducted on a number of five channels with ~1 mm^2^ GSA, 500 μm-wide rectangular carbon contacts, and one stainless-steel circular ring of ~2 mm^2^ GSA, which is the original stainless-steel macrocontact of the bipolar microelectrode. Note that it was not among the goals of this study to thoroughly compare the merits of the carbonic material against the original macrocontact material. Figure 7c,d illustrate the quasi-linear evolutions to the current amplitude of both the extracted driving voltage half-ranges (i.e., *(V_A_ − V_C_)/2*) and of the estimated V_acc_ for the analyzed electrodes. Following the waveform corrections with the access voltages, the voltage excursions relevant for the drop across the electrode-electrolyte interface (interfacial polarization ranges) are represented in Figure 7e,f, where the CV-deduced cathodic and anodic limits of the water window for the carbonic paste electrodes are also superposed. It can be noticed that, for these current pulse parameters, 9 mA amplitude current stimulation can be safely performed through these electrodes, and the CIC for the carbonic electrodes is at least 135 μC/cm^2^. However, these results must be taken cautiously. Further experimental validation will be needed, given the difference between the real, in vivo, chemo-physics and the in vitro, free-electrolyte analysis conducted here.

As mentioned, it was not in the scope of this study to benchmark these performances against those of the materials generally used as macrocontacts in clinical bipolar microelectrodes (e.g., stainless steel). However, the comparative results presented here for the 2 mm^2^ GSA stainless-steel contact (which, up to some safe corrosion limits, is also a quasi-capacitive charge injection material [24], owing to a thin superficial oxide layer) indicate a slightly lower charge injection performance than that of the graphitic material, but which still covers very well the acute clinical scope. PtIr is known to have a pseudocapacitive charge transfer, with an electrolysis window of [−0.6, +0.8] V and a charge injection limit of 100–150 μC/cm^2^ for 200 μs cathodic-first pulses in PBS and at GSAs comparable with this work, that can be increased by applying an anodic bias between pulses [39].

### 3.5. In Vivo Experiments

The targeted rat hippocampal (HC) and thalamic (TH) regions for LFP sensing and test macrostimulation were planned to be centered in the following positions: HC: −2.4/2.0/3.3; TH: −2.4/2.0/5.3 (coordinates from bregma, in mm, antero-posterior/medio-lateral/dorso-ventral). For each position, the distal tip of the probes reached ventrally lower (~2–3 mm) to account for the axial offset to the carbonic contacts.

Before insertions, each channel contact of the three probes employed was pulse-activated in PBS solution (see Supplementary Materials for details on the electrode preparation).

#### 3.5.1. Macro-Recording Tests

**Figure 8:**
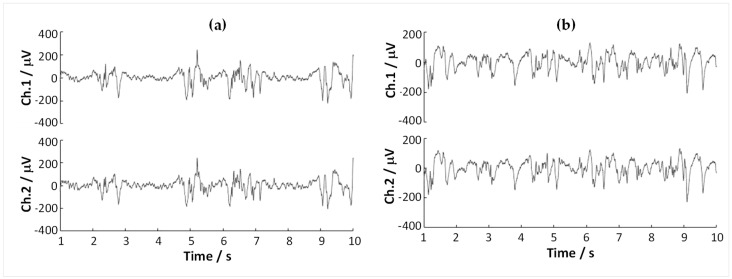
LFP recordings using 2-channel carbonic segmented contacts at 1 kHz sampling rate, using a DC-coupled amplifier with gain ×24. The data were bandpass filtered to 1–35 Hz (3rd order Butterworth) and notch filtered to remove the 50 Hz noise. (**a**) Hippocampal position. (**b**) Thalamic position.

LFP recordings were performed for each segmented carbonic contact in both the HC and TH positions. Figure 8 presents typical records during the experiment. While a one-to-one comparison in the sensing quality of the standard stainless-steel macro-electrode and the segmented contacts was not within the scope of this study—due in part to the limitation of having relatively thick probe shanks simultaneously in the rodent brain and close proximity to each other—one can observe that the segmented contacts accurately reflect the expected LFP patterns in the presence of deep anesthesia: in HC positions, burst-suppression patterns [44] could be frequently encountered (Figure 8a). Note that the small differences sensed by the two channels can be observed at a close examination; these near similarities indicate that in this experiment, the electrodes are not sensing the border areas of the functional regions. However, in the real neurosurgical application, the targeting function of the electrodes consists precisely in detecting the higher differences between the sensing channels that may be caused when the electrode’s trajectory passes at the frontier of the targeted DBS functional nucleus. These data suggest that the segmented carbonic contacts can record LFPs directionally and with high signal quality, supporting the in vitro results presented in the Supplementary Information (Figure S12).

#### 3.5.2. Macrostimulation Tests

**Figure 9:**
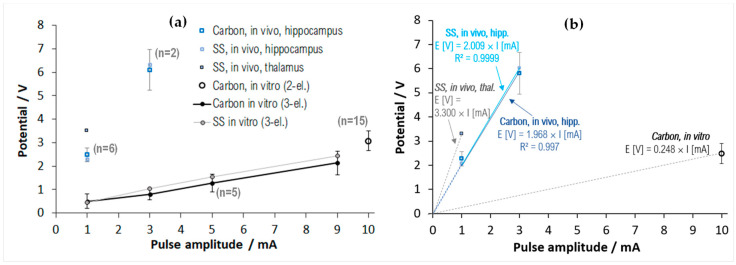
(**a**) Driving voltage half-ranges ((V_A_ − V_C_)/2): comparative overlay of two-electrode in vivo and in vitro measurements, with the three-electrode pulse chronopotentiometry data (Figure 7c), and with 2-electrode pulse test measurements performed in vitro for various carbon-terminated electrodes. The geometric surface area was 1 mm^2^ for carbon electrode contacts (“Carbon”) and 2 mm^2^ for the stainless-steel (“SS”) contact. Signal sampling was 31.25 μs for the 2-electrode data and 1 μs for the 3-electrode data. In the 2-electrode measurements, the reference was a stainless-steel needle. The error bars correspond to the standard deviation (*n* > 1 only for carbonic contacts). (**b**) Estimated corresponding ohmic contributions (V_acc_) to the total polarization—under a purely ohmic overpolarization hypothesis.

During the stimulation tests, the 32 kHz sampled driving voltage transients (V_C_ to V_A_) showed a significant amplitude increase when delivering 1 and 3 mA amplitude pulses, if compared with the transients recorded during the in vitro pulse chronopotentiometry tests. Figure 9a displays a comparative overlay with the 3-electrode in vitro chronopotentiometry data presented in Figure 7c and with 2-electrode pulse test measurements performed in vitro for a number of 15 carbon-terminated electrode probes. Notwithstanding the experimental differences—in measurement configurations and sampling conditions—the notable increases in vivo vs. in vitro represent an essential issue discussed in various electrode materials and application contexts [24,25,27,45,46,47,48].

#### 3.5.3. Overpotential Analysis

There are several offending factors presumed to lead to the expansion of the driving voltage observed when comparing in vivo with in vitro pulsed current stimulation experiments, such as unrealistic composition and properties of the electrolyte used for the analysis; biofouling at the electrode surface (encapsulation with a layer of cells and proteins); porous-tortuous morphology of the biologic environment leading to less efficient diffusion of the ionic charge carriers; chemical changes in the vicinity of the electrode contact; and the temperature of the living tissue. According to the type and properties of the tissular environment, as well as the other experimental specifics (primarily, the material of the electrode and the associated balance of its capacitive-faradaic charge transfer, and the extent of the time spent in the tissue), these factors contribute with various weights to the large increase observed in various experiments; for example, doped nanodiamond electrodes were shown to be immune to biofouling when subcutaneously implanted for 6 weeks in rat models, contrary to the performance of TiN electrodes [27], confirming this well-known quality of carbon-based electrodes.

Concerning this issue, the immediate priority is to determine whether the increased voltage excursions also incorporate an unsafe rise of the polarization range across the electrode–electrolyte interface itself (i.e., E_C_ = V_C_ + V_acc_ to E_A_ = V_A_ − V_acc_), as this would mean that the current injection at the level needed to elicit the desired therapeutic effect may engage an electrode-level overpotential that reaches the electrolysis limits [45]. This is not an easy evaluation to be performed, as it requires either (pseudo)3-electrode [27,46,48] electrochemical measurements performed in vivo at high-enough sampling rates or complex model-based estimations [49,50]. One secondary concern caused by the in vivo expansion of the total polarization is related to the mechanical stability of the electrode when subjected to the enlarged total power; for example, activated iridium oxide film (AIROF) electrodes of 2000 μm^2^ GSA lowered for 500 μm into the brain of a bird model showed severe delamination of AIROF when the driving voltage of the 300 μs cathodic pulse was increased to 4 V to compensate for the in vivo-occurred access voltage surplus, and thus support a 50 μA current pulse [46]. In the third place, after the first two concerns are surmounted, the driving voltage expansion can still cause intraoperative technical impediments if the voltage reaches the compliance limit of the electronics inside the clinical system.

Referring to the in vivo experimental data analyzed here, their scarcity—imposed by the need to minimize the anesthesia time and to protect the tissue by slow and careful insertion/extraction—inherently restricts the basis for firm statistics on the polarization surplus. However, the data have good physical consistency: the carbonic and stainless-steel (SS) potentials in hippocampal positions correlate well, and the PBS-measured 2-electrode potentials over a large population (*n* = 15) also correlate well with the former pulse chronopotentiometry data (*n* = 5). As the data collected during the experiment are sparsely sampled (31.25 μs), it is also difficult to draw a firm conclusion concerning the polarization surplus falling on the electrode–electrolyte interface at the locations inside the brain tissue (the primary concern mentioned above). Within these limitations, we have chosen to test the presumption that the in vivo-occurring voltage surplus does not fall on the electrode interface (i.e., it has an ohmic character due exclusively to the tissular environment); that is, one could deduce that ohmic drop just by subtracting the interfacial polarizations that were measured in vitro from the total polarizations measured in vivo, according to:

0.5·(V_A_*^in vivo^*–V_C_*^in vivo^*) − 0.5·(E_A_*^in vitro^*–E_C_*^in vitro^*) = R_acc_·I (1)

Under this hypothesis—that will be further analyzed in the next section—after subtracting from the 2-electrode data of Figure 9a the estimated half-ranges of the total interfacial polarization—calculated from the cumulative data presented in Figure 7e—one arrives at the result plotted in Figure 9b; the slopes towards the origin are also calculated on the plot, providing the estimated access resistances R_acc_. It results that in hippocampal positions, R_acc_ for both materials is centered at ~2 kΩ; in the thalamic position, R_acc_ increases to ~3.3 kΩ; while in PBS, the average R_acc_ overall electrode specimens is centered at ~250 Ω. These values validate well with the 1 kHz impedance measurements that are aggregated in Table 2, as well as with the in vitro data (Supplementary Table S2).

To evaluate the degree to which the temporarily adsorbed biofilm has a role in the driving voltage and impedance increase, after removal at the end of the in vivo procedure, the electrodes were immediately reanalyzed in PBS solution *without pre-washing*. The measured 1 kHz impedances showed a 20–40% increase, and the driving voltage half-ranges for 10 mA pulse amplitude averaged 10–20% higher than the pre-insertion measurements. These changes could be, indeed, due to the organic residues that adhered on the surface, representing an obstructive film to the current flow; however, their extent is much smaller than what is noticed in Figure 9a and Table 2, implying that the observed effect has additional sources.

**Figure 10:**
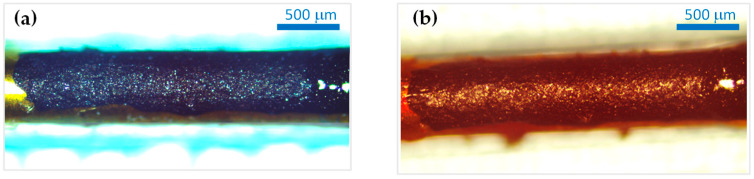
Representative images for a carbonic contact: (**a**) before the in vivo experiment and (**b**) after the experiment (unwashed).

Optical microscopy images, taken before and after the in vivo experiments, indicate that no optically apparent surface morphology changes occurred during the procedures (exemplification in Figure 10).

### 3.6. Emulative Experiment for the In Vivo Overpotential

We have enumerated the physico-chemical factors that contribute in various proportions to the significant increase in the driving voltage transients when the electrodes are immersed in a living tissue environment vs. the in vitro evaluations. With one exception—i.e., the composition and properties of the electrolyte used for the analysis [45,51]—we presume that the connecting label for all the other factors is the change of the ion transport (diffusion) kinetics through the electrolyte and near the electrode contact.

The factors interfering against the interelectrode charge transport contribute to generating irreversibility, leading to expansions of the voltage drops along the pathway; the resulting voltage expansion is synonymously called overpotential, (over)polarization, and overvoltage. The ion diffusion kinetics directly impacts this voltage outgrowth by deciding the available rates for interelectrode transport (i.e., mobility), and for various chemo-physical mechanisms at the electrode, such as redox reactions (faradaic charge transfer), or just charge accumulation (capacitive charge transfer).

Understanding and using optimally the underlying chemo-physics of these connected processes is an area of great interest for research in energy storage and conversion technologies, such as batteries [52,53], and fuel cells [54,55], where various modeling approaches have been developed to account for the modified ion conduction properties in porous-tortuous path structures (see Supplementary Materials for additional literature details). Considerations of these sorts can be easily applied to the specifics of the application addressed in the present work.

Indeed, the overpotential growth observed in the in vivo studies has to do with one or more of the conventional components of the polarization [25,55]: *ohmic* drop (caused by the ionic resistance of the electrolyte and by the non-ionic electrical resistances of the pathway), *activation, or charge transfer* (corresponding to the energy needed in the faradaic charge transfer), *concentration, or diffusion* (determined by the concentration differences of the electroactive species between the electrode surface and the bulk electrolyte, reflecting an insufficient supply thereof). Considering the assessed capacitive behavior of the carbonic material used in this work, it is adequate to neglect here the role of the activation polarization, leaving the diffusion-type of polarization as the main culprit for the polarization outgrowth observed in vivo.

Inspired by the studies sampled above, we conducted a simple experiment investigating the effect of deliberately restricting the ionic diffusion transport while performing pulse chronopotentiometry measurements in PBS solution. The model consists of interposing a spongiform obstructing sheath on the ionic pathway towards the electrode, mimicking a diffusion-restrictive environment that attenuates the ionic mobility. We mention that a modeling approach of this type was first used in [56], where the impedance variation in cochlear stimulating implants due to cellular encapsulation was modeled in vitro using a monolayer of cells grown over gold electrodes.

As a heavily packed, complex assembly of nerve and glial cells, the nervous tissue can be seen, indeed, as a highly dense, highly anisotropic fibrous-porous material [57,58,59] that offers only tortuously long ionic passage channels between the electrode and the counter-contact (see Supplementary Materials for additional literature details).

**Figure 11:**
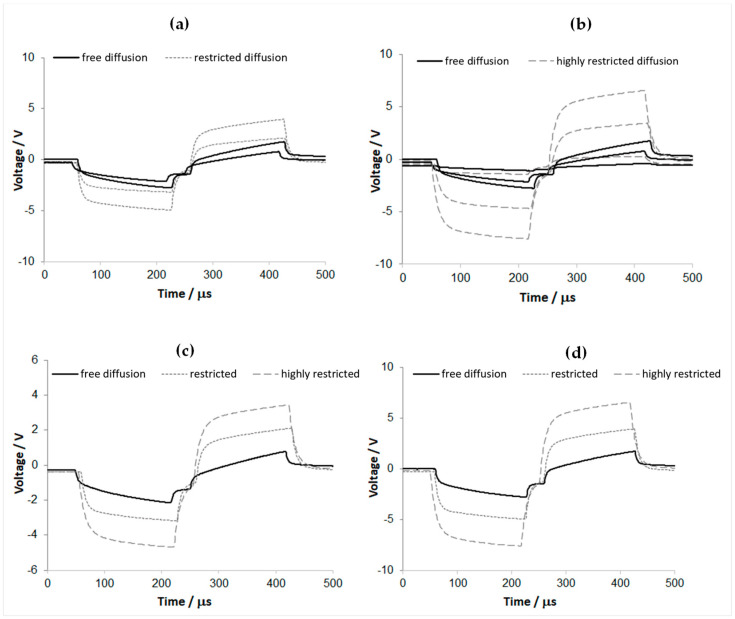
Driving voltage transients measured in the chronopotentiometry experiment (150 Hz biphasic pulses, 150 μs phase duration) for one 1 mm^2^ GSA carbonic contact in PBS solution, for various current pulse amplitudes, and various levels of diffusive restriction: (**a**) 5 and 9 mA elicited voltage transients, for free diffusion and medium restriction, (**b**) 1, 5 and 9 mA elicited transients, for free diffusion and large restriction, (**c**) 5 mA, and (**d**) 9 mA elicited transients, for three levels of diffusive restriction. Sampling interval: 1 μs.

The experiment consisted of covering one of the carbonic contacts fabricated in planar geometry with a spongiform material (Figure S13) and fastening it to the support glass slide with parafilm tape to obtain a firm coverage on and around the carbonic contact while leaving open the proximal and distal ends for electrolyte access. Two different levels of fastening strength were used to simulate the effects of different porosity-tortuosity or diffusion restrictions. Three-electrode chronopotentiometry was performed in PBS solution, in the conditions described earlier in the text, for both these diffusion restriction levels. As shown in Figure 11, the overpotential surplus caused by the restricted diffusion is significant and depends on the sponge tightening level (i.e., the highest tightening for highly restricted diffusion). One also notices that the voltage profiles over the pulse interval are evolving capacitively, and they keep good parallelism with the free diffusion profile, indicating reversible (non-faradaic) charge transfer for both levels of diffusive restriction and up to 9 mA pulse amplitude (135 μC/cm^2^).

**Figure 12:**
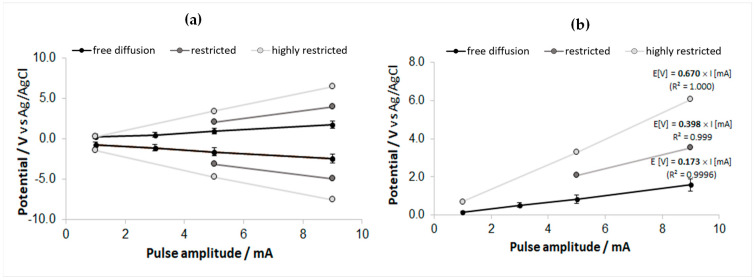
Comparative overlay (with the free diffusion data used in Figure 7c,d) of the results obtained from pulse chronopotentiometry measurements (150 Hz biphasic pulses, 150 μs phase) for 1 mm^2^ GSA carbon-contact electrode in PBS solution, subjected to two levels of diffusive restriction. The error bars correspond to the standard deviation (*n* = 5). (**a**) Cathodic and anodic potential peaks (raw, uncorrected for access voltages, i.e., V_C_ and V_A_) vs. the current pulse amplitude. (**b**) The access voltages V_acc_, evaluated from the transient waveforms vs. the current pulse amplitude; the linear fits indicate the estimated access resistances of 173 Ω for unobstructed diffusion, 398 Ω for moderately restricted diffusion, and 670 Ω for the largest diffusive restriction.

Figure 12a presents an overlay of the pulse-amplitude dependence for the measured cathodic and anodic voltage extremes (V_C_ and V_A_) at the two levels of diffusion restriction, with the former cumulative data for *n* = 5 free diffusion cases. In contrast, the overlay in Figure 12b correspondingly compares the evaluated access voltages. One can observe very linear evolutions to the current amplitude in both displays. The effects of the progressive level of diffusive restriction are also manifest, and the linear fit through the axes’ origin, presented in Figure 12b, indicates an ~4× increase in the access resistance between the free diffusion case and the largest diffusive restriction case. Comparing the plots in Figure 12b with the equivalent in vivo counterparts presented in Figure 9b, one notices that the estimated slopes of the hippocampal and thalamic V_acc_ vs. I characteristics differ only in scale from these in vitro results. Moreover, the difference between the access resistance in the thalamic and hippocampal electrode positions may support the present model if we consider the significant morphological packing contrast between these regions: from a comprehensive review of the current knowledge about cell densities and morphologies in the rat brain [60], one can deduce that the total cell density (neurons, astrocytes, and microglia) in the thalamus is at least 4-times higher than in the hippocampus (i.e., ~200,000/mm^3^, vs. ~50,000/mm^3^). A further overlay (Figure S14) compares the V_acc_-corrected transient maxima for the in vitro-measured free diffusion case vs. the diffusion-restricted cases (following corresponding subtraction of Figure 12a,b data plots). Even though the statistics of the underlying data are insufficient to draw a firm conclusion, the differences—including the rise of the inter-pulse potential for the diffusion-restricted situations—observed in these comparative plots of “residual” polarizations may indicate that the restricted diffusion that was modeled here may entail a more complex phenomenology than what could be reproduced by a purely ohmic potential addition.

The ionic mobility-reducing mode was tested on planar electrodes; still, according to the constantly observed functional equivalences between the two geometries, it is equally relevant for the actual cylindrical geometry. We believe that the results presented here indicate that this modeling path, and associated data processing, may be combined successfully with the path of optimizing the composition and properties of the electrolyte used for the analysis, to provide more accurate immediate insight on the expected performance and safety of acute and chronic stimulating electrodes.

## 4. Discussion—Limitations of the Study

This work is at the stage of feasibility and demonstration study, and the main points that were planned to be addressed were the fabrication of segmented recording–stimulation electrode channels on the ridge of a long and thin, electrically insulated, stainless-steel shank; and the firsthand characterization of the graphitic paste material as a contact electrode. On the development scale of such an invasive device, this current stage is just incipient. To ensure total safety and viability, several improvements, studies, and tests need to be performed based on the problems observed. We provide here a short list of these problems and next-step needs.

Regarding the fabrication of the electrode probe, multi-axis automated depositions of the functional materials will improve the throughput, overall quality, and reproducibility. While increasing the number of radial views (number of channels) is possible with the tools employed here, we envisage reducing the fabrication complexity using these novel printing tools and methods. The described execution of the segmented conductive channels and the stacked distal functional contacts required strained procedures for precise alignments and rotations. This led to fabrication errors and removals from the testing pool. Proximal wire contacting was kept as basic as possible, which led to no faults, but multipin-based contacting is needed for real integration. The outer insulation needs to be significantly improved; this process should focus on (i) the identification of an easily processable, biomedical-grade coating material or a shrink-polyimide microtube like the one used for the inner insulation of the original microelectrode; and (ii) a better control of the distal position of the insulation limit, to provide precise delineation of the exposed electrode contacts.

The biocompatibility profile must be further evaluated to assess the functional carbonic material. More systematic mechanical stability tests, such as agar-phantom penetrations, should also be performed for thorough evaluations of cast residues, morphology, and electrochemical change effects. Although the variability used for some of the experimental parameters (e.g., potential windows of CV scans and of electrochemical activations, GSAs) was somewhat helpful at this stage for providing a better, multi-view insight into the material behavior, in the subsequent development stage, the CV and EIS results need to be both stabilized and brought to higher statistical consistency. It is expected that the process automation planned for the next development step will both improve the statistical quality—by enhancing fabrication tolerances (deposition thicknesses, widths, and limits)—and provide for larger sample sizes for better validation statistics. Furthermore, the safety and endurance validation processes need more data to be collected and analyzed, e.g., at various pulse durations, restricted diffusion levels, and electrolyte compositions (including pH and temperature control). The in vitro sensing tests must be conducted systematically to evaluate the distortion profile. Also, further in vivo characterization experiments need to be performed using a (pseudo)3-electrode-based configuration to support the process of developing solid answers on the interfacial phenomenology of the carbonic material.

## 5. Conclusions

Electrophysiological mapping probes used in functional neurosurgery require functional broadening to optimize the target refinement. Our paper presents the first-stage development and characterization of circumferentially segmented probe channels for intraoperative directional sensing (recording) of local field potentials and directional macrostimulation, thus supplementing the existing capabilities of a typical long (230 mm), thin (550–770 μm diameter), and semirigid clinical probe. At this stage, it has been shown by various in vitro and in vivo characterizations that this probe enhancement is effective. Full validation of these functional enhancements needs further process and characterization refinements.

The exposed functional contacts are fabricated from a graphitic paste with convenient properties, such as predominantly capacitive charge transfer at a large and symmetric water window ([−1.5, +1.2] V versus Ag|AgCl), excellent processability and adhesion strength, mechanical, and electrical endurance. The segmented, 1 mm^2^ GSA, rectangular electrode contacts provide a conservative limit of charge injection of 135 μC/cm^2^ for 9 mA amplitude biphasic pulses of 150 μs phase interval, 150 Hz frequency, and no voltage bias, thus covering the operational demands during test stimulation for intraoperative electrophysiological mapping.

An ionic mobility-reducing model was proposed and analyzed, as a partial explanation of the inherently modified behavior—in terms of driving voltage transients—that appears in the nervous tissue environment.

## 6. Patents

The underlying patent application of this work is Popa, R.C.; Buiu, O.; Avram, M.A.; Şerban, C.A.; Barborica, A. Probe with circumferentially segmented multielectrodes of electrically conductive carbon film, to be used in multi-channel neurophysiological exploration and process for carrying out the same, RO Patent 134,873 A2 RO-BOPI 4/2021 (https://patents.google.com/patent/RO134873A2/en).

## Figures and Tables

**Table 1:** Comparative EIS-extracted parameters for probe channels in planar (P1, P2, P3), and cylindrical (Ca, Cb) substrate geometries. The fitting equivalent circuit is presented in Figure 4.

Probe Channel	R_s_ (Ω·cm^2^)	CPE-Q_0_ (S·s^n^·cm^−2^)	CPE-n	R (Ω·cm^2^)	C (F·cm^−2^)	Fitting χ^2^	|Z|_EEI-1kHz_ (Ω)	|Z|_EEI-10Hz_ (Ω)
P1—plane P2—plane P3—plane	1.0	1.1 × 10^−4^	0.71	0.6	3.7 × 10^−5^	2.4 × 10^−3^	275	25006
	0.9	1.0 × 10^−4^	0.69	0.8	2.8 × 10^−5^	3.3 × 10^−3^	383	31466
	1.4	2.3 × 10^−4^	0.77	1.3	2.8 × 10^−5^	2.6 × 10^−3^	190	14596
Ca—cylinder Cb—cylinder	0.3	1.0 × 10^−4^	0.54	2.9	0.9 × 10^−5^	5.0 × 10^−3^	1273	67663
	0.8	0.9 × 10^−4^	0.65	1.3	1.4 × 10^−5^	3.6 × 10^−3^	740	46081

**Table 2:** Impedances (Ω) measured at 1 kHz during the experiment using the Guideline 5 system (2-electrode configuration).

	Carbon, In Vitro-*PBS* *n* = 6	Carbon, In Vivo *Hippocamp.*, *n* = 6	SS, In Vivo *Hippocamp.*, *n* = 1	SS, In Vivo *Thalamus*, *n* = 1
Average Std. dev.	705	3353	2670	3950
	125	513	-	-

## Data Availability

The data presented in this study are available on request from the corresponding author.

## Supplementary Materials

The following supporting information can be downloaded at: https://www.mdpi.com/article/10.3390/s23177497/s1.
