# Supplementary material for: Functional Enhancement and Characterization of an Electrophysiological Mapping Electrode Probe with Carbonic, Directional Macrocontacts
Source: Sensors (Basel). 2023 Aug 29;23(17):7497. doi: 10.3390/s23177497 (PMC10490806; doi:10.3390/s23177497)
Supplement: Supplementary file 1 [file sensors-23-07497-s001.zip › sensors-2550487-supplementary.pdf]

Supplementary Material

Functional Enhancement and Characterization of an Electrophysiological Mapping Electrode Probe with Carbonic, Directional Macrocontacts

Radu C. Popa [1,*], Cosmin-Andrei Serban [2,3,4], Andrei Barborica [2,3,4], Ana-Maria Zagrean [5], Octavian Buiu [1], Niculae Dumbravescu [1], Alexandru-Catalin Paslaru [5], Cosmin Obreja [1], Cristina Pachiu [1], Marius Stoian [1], Catalin Marculescu [1], Antonio Radoi [1], Silviu Vulpe [1] and Marian Ion [1]

[1] National Institute for R&D in Microtechnologies–IMT Bucharest, 077190 Bucharest, Romania; octavian.buiu@imt.ro (O.B.); nicky_dumbravescu@yahoo.com (N.D.); cosmin.obreja@imt.ro (C.O.); cristina.pachiu@imt.ro (C.P.); marius.stoian@imt.ro (M.S.); catalin.marculescu@imt.ro (C.M.); antonio.radoi@imt.ro (A.R.); silviu.vulpe@imt.ro (S.V.); marian.ion@imt.ro (M.I.)

[2] Termobit Prod Srl, 020281 Bucharest, Romania; cserban@fh-co.com (C.-A.S.); abarborica@fh-co.com (A.B.)

[3] Fhc, Inc., Bowdoin, ME 04287, USA

[4] Faculty of Physics, University of Bucharest, 077125 Magurele, Romania

[5] Physiology and Neuroscience Department, "Carol Davila" University of Medicine and Pharmacy, 050474 Bucharest, Romania; ana-maria.zagrean@umfcd.ro (A.-M.Z.); catalin.paslaru@umfcd.ro (A.-C.P.)

* Correspondence: radu.popa@imt.ro; Tel.: +40-21-269-0770 (ext. 219)

1. Characterization of the carbonic material—*Morphostructural analysis*

The JELCON CH-8 paste was developed by the manufacturer for use in membrane switches and flexible printed circuits and was specified as offering excellent flexibility and compatibility with silver paste. At this development stage, the paste was not tested for long-term biocompatibility; however, its post-curing (at 140 °C, 20 minutes) structural and composition analysis showed a high-quality graphitic content. The paste has a thixotropic viscosity, specified as 23500-36500 cPs. The cured material proved to have very good mechanical abrasion resistance (as tested by manual power-rubbing against plastic syringe tips wrapped in one layer of lab paper sheet, both in dry and wet conditions) and offers the highest adhesion class (cross-hatch ratings 5B, as measured using an ASTM D-3359 dedicated kit) both to the Kapton® foil substrate and to the cured silver inks.

SEM, XRD, and Raman analyses were performed on 1 × 1 cm Kapton® foil substrate coated with ~100 μm thickness (estimated "on wet", by the thickness of the screen mask employed) of thermally cured carbonic paste.

Primary adhesion tests were performed with an ASTM D-3359 dedicated paint adhesion test kit to determine the cross-hatch adhesion rating. A few drops from the Ag NP inks or from undiluted and diluted carbonic material were dispersed using a spatula and lateral 100 μm thick spacers over a piece of isopropyl alcohol cleaned and plasma-pen-treated Kapton® foil. The coating was then thermally cured according to the producer's specifications and the cross-hatch cutting, tape application, and visual assessment were performed as specified in the standard.

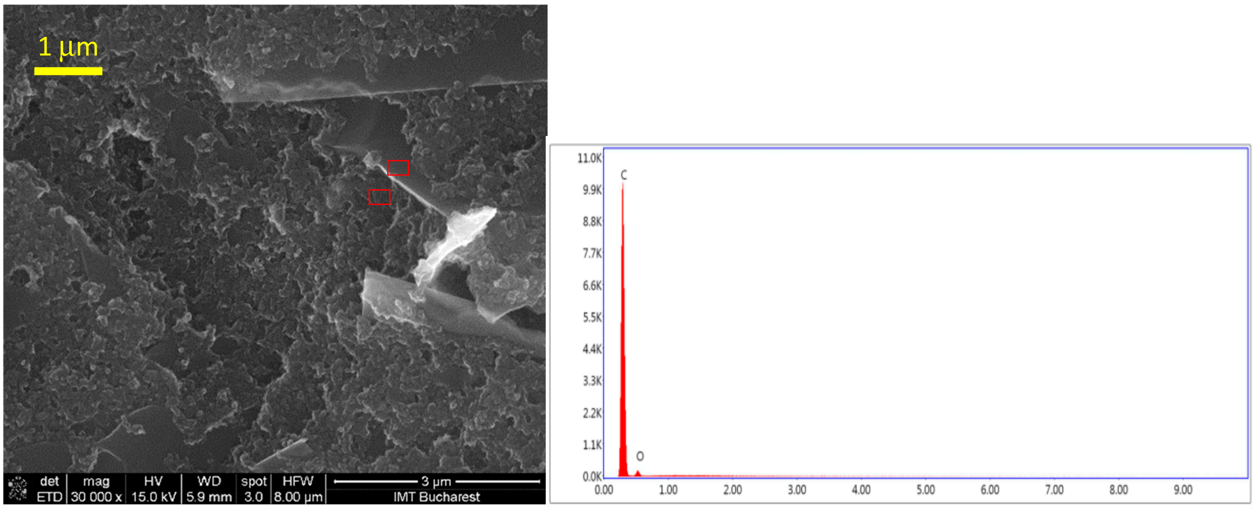

Figure S1. SEM image—left—and EDX elemental analysis (at 15 keV)—right—of the thermally cured carbonic paste. The elemental analysis showed that the areas marked by red rectangles—on graphitic lamella and off graphitic lamella—contain very similar carbon and oxygen contents (94% vs. 92% C and 6% vs. 8% O).

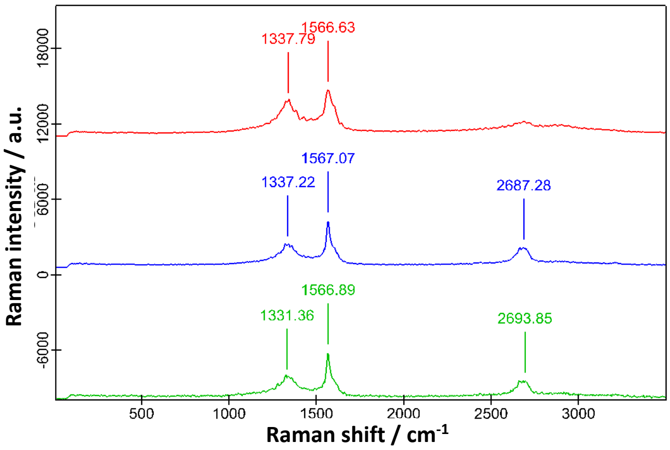

Figure S2. Raman spectra of the cured carbonic material deposited on polyimide (Kapton® foil) collected on various points of the surface.

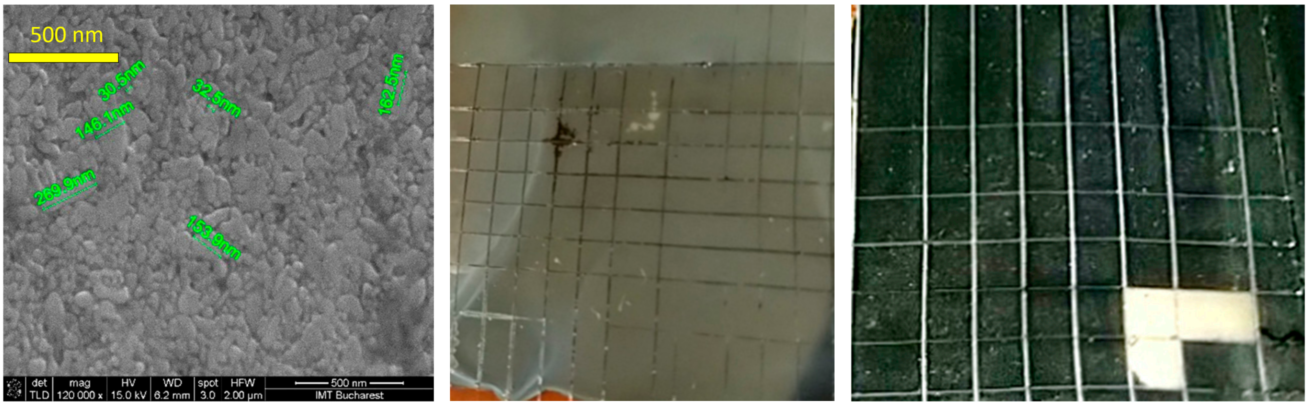

Figure S3. *Left*: Typical SEM image of thermally cured Ag NP ink, at ~50 μm thickness (120 000 × magnification). *Center*: Cross-hatching test per ASTM D-3359 for cured Ag NP ink on polyimide (Kapton®) foil substrate. *Right*: Cross-hatching test per ASTM D-3359 for cured carbon paste/Ag NP ink/polyimide substrate.

2. Characterization of the carbonic material—*Electrochemical impedance spectroscopy*

Modeling parameters

The constant phase element is a synthetical equivalent of specific physicochemical processes—such as a non-ideal capacitance of the electrochemical double layer—and has a complex-valued impedance represented as $Z_{CPE} = Q_0^{-1} \cdot (j\omega)^{-n}$, where j is the imaginary number and ω is the angular frequency, while Q_0 (the CPE magnitude) and n (the CPE power, with $0 < n < 1$) are the tunable parameters of the model; this equivalates to an R(ω)-C(ω) frequency-dependent series circuit with $R(\omega) = Q_0^{-1} \cdot \omega^{-n} \cdot \cos(n\pi/2)$ and $C(\omega) = Q_0 \cdot \omega^{n-1} \cdot \sin^{-1}(n\pi/2)$, and tends to a plain resistor when n approaches 0, or to a plain capacitor when n approaches 1.

Supplementary Table S1. Specific EIS modelling parameters for the undiluted, and diluted carbonic paste, resulted by fitting the experimental data to the best equivalent circuit (presented to the right). The data indicate similar electrochemical mechanisms for the two forms.

	R_s ($\Omega \cdot cm^2$)	CPE-Q_0 ($S \cdot s^n \cdot cm^{-2}$)	CPE-n	R ($\Omega \cdot cm^2$)	C ($F \cdot cm^{-2}$)	Fitting χ^2
**Undiluted**	3.9	2.1×10^{-3}	0.88	10.5	3.8×10^{-4}	3.7×10^{-3}
**Diluted**	25.7	9.1×10^{-4}	0.75	34.8	9.0×10^{-4}	2.9×10^{-3}

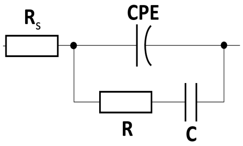

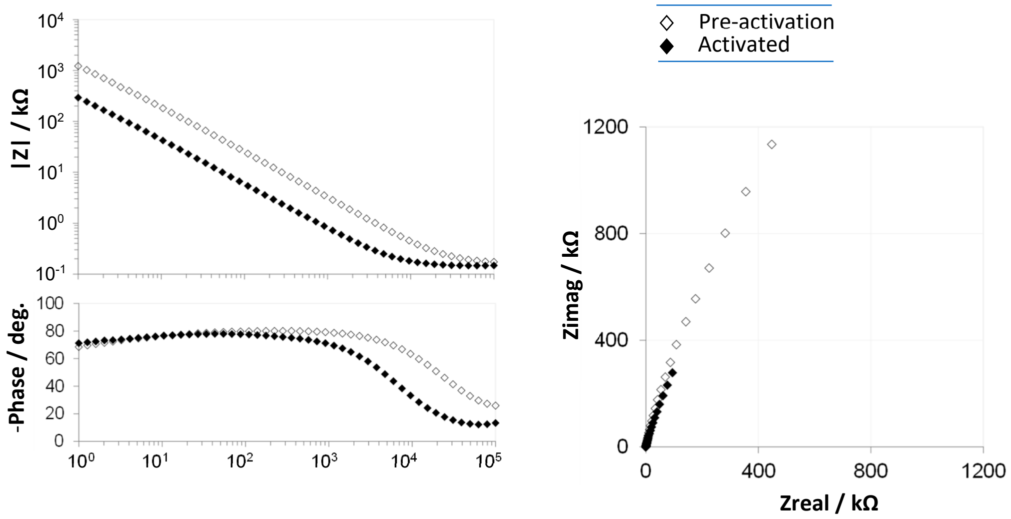

Figure S4. Example of EIS Bode and Nyquist plots before, and after carbon surface activation. Diluted carbonic paste, GSA: 1 mm^2, activation by pulses.

3. Planar substrate probes—*Supplementary Methods*

Printing the Ag NP ink lines and the carbonic paste contacts required optimization steps to obtain the designed widths, uniformities, and electrical resistance. The inkjet-printed lines were optimized, according to the ink type, at 250-300 μm width and 2-3 μm thickness after curing, resulting in a linear resistance of 1.5-2 Ω/cm. This was realized by employing initial platen angle calibration, single-nozzle jetting, ink producers' recommended jetting waveforms and printhead

temperatures, 20-50 μm drop-spacing, 50-60°C platen temperature, and multiple paths (15-25) with interlayer delays (3-5 min.) for full drying between the layers.

For the extrusion printing of the graphitic paste stacked over the Ag NP lines, luer-lock syringes with 150 μm diameter needles were used, and the extrusion was performed at 80 psi (5.5 bar) and 200-300 mm/min movement speed. A particular quality enhancement of the printed shapes—that were targeted as ~500 μm wide and ~3000 μm long rounded rectangles centered above the 250-300 μm wide silver route—was attained by optimizing an especially designed displacement routine, whereby the targeted rectangle width was achieved from three parallel passages in the axial direction of the printing head; for process transfer to the thin cylindrical geometry, the exterior passages were adjusted to ensure symmetric height differences to accommodate for the cylindrical surface and for the lateral material spread. The height of the cured carbonic shapes is 12-15 μm, and they were measured to add about 7-10 Ω to the overall resistance of an Ag-carbon route.

Figures S5 and S6 illustrate the configuration of the planar electrode mockups.

(a) (b)

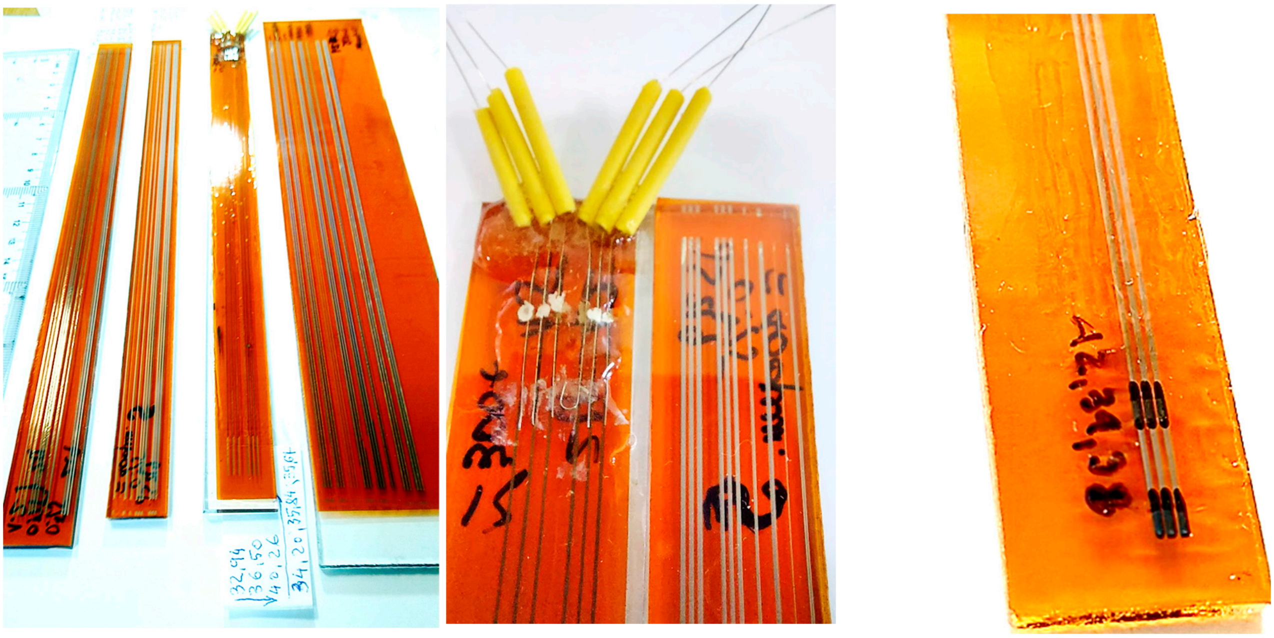

Figure S5. Planar electrode mockups on 250 mm long glass slides. **(a)** Closely spaced Ag NP ink routes inkjet printed on Kapton® foil. Widths: 250-500 μm. Lengths: 230 mm. Interdistance: 200-500 μm. **(b)** Electrode routes with print trials for the carbonic contacts.

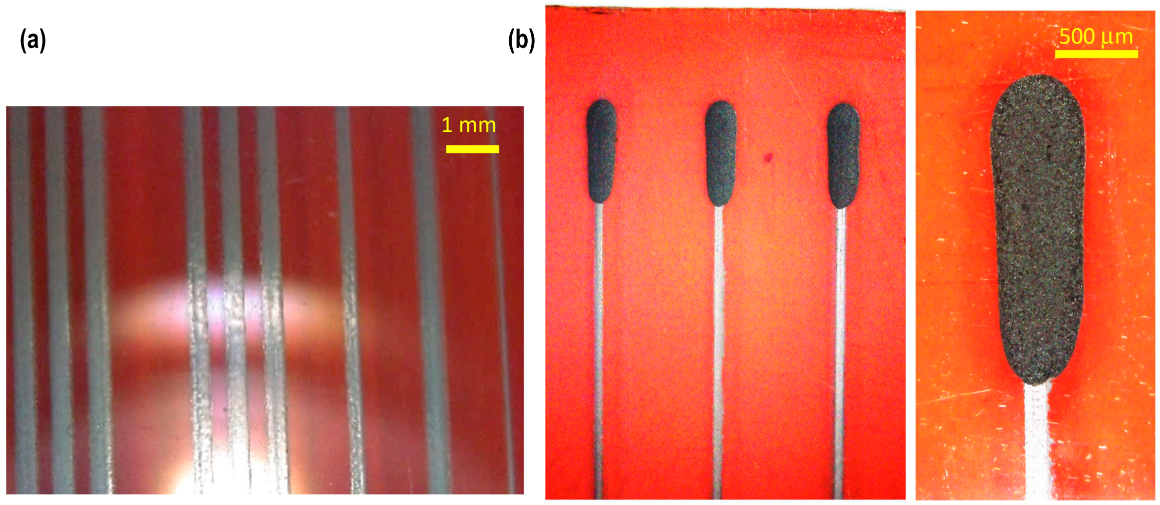

Figure S6. (a) Ag NP ink traces realized in planar substrate geometry by inkjet printing. Widths: 250-300 µm. Length: 230 mm. (b) Planar electrodes formed by carbonic contacts stacked over Ag NP ink traces, realized by extrusion printing. Width: 500 µm. Length: 2000 µm. *Right*: detailed view.

4. Planar substrate probes—*Frequency-related effects*

CV measurements performed for the planar electrodes after their electrochemical activation and exterior electrical insulation revealed that at a slow scan rate (50 mV/s) the voltammograms may exhibit a surface confined chemically reversible couple (Figure S7), like the ones attributed to silver contamination in [S1].

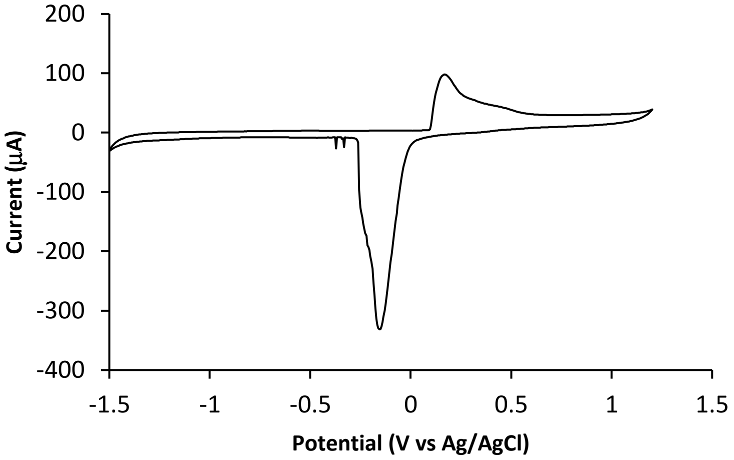

Figure S7. Slow-rate voltammogram (50 mV/s) for a 1 mm^2 GSA carbonic paste deposition contacted with Ag NP ink printed underneath after electrochemical activation between -2 and +2 V (at 100 mV/s).

We investigated this matter through a specific experiment. Starting with a freshly fabricated electrode (carbonic deposition of 1 mm^2 GSA, with no initial electrochemical activation), a succession of CV scans was performed, from high-rate (10 V/s) towards slow-rate (50 mV/s), while alternating the potential range between [-1.5, +1.2] V and [-2, +2] V. The relevant scans are presented in chronology in Figure S8.

It can be noticed that the first emergence of the silver redox couple occurs at step no. 6, performed at 100 mV/s. Subsequently, after performing the large-range scan at 50 mV/s (step

no. 7), the next low-range scan (step no. 8) shows a very pronounced peak pair. Like observed in [S1], too, the water window was not affected by this occurrence. EIS measurements made after scans 6 and 8 showed that the corrected impedance (by subtracting the series ohmic resistance as determined from the best-fit model) has a significant decrease, especially at low frequencies, e.g., at 10 Hz, from 4.4 kΩ to 719 Ω.

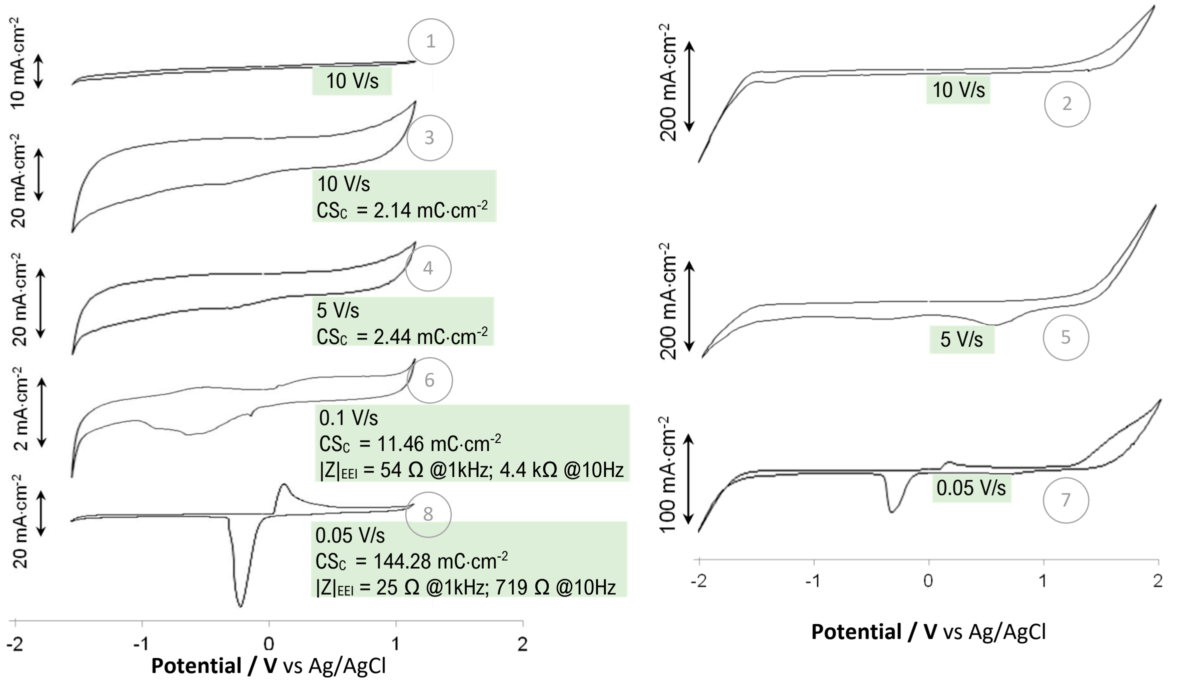

Figure S8. Successive high-to-low scan-rate voltammograms, starting from a freshly-deposited (diluted) carbon paste electrode (GSA: 1 mm^2) contacted by Ag NP ink printed underneath. The experimental succession is indicated by the circled numbers (1 to 8).

These results confirm the gradually increased penetration depth [S2] of the ionic current into the pores of the material with the decrease in the sweep rate. Prompted by the reduction in the scan rate, as well as by the enforced ionic driving during the large-range scans, the faradaic processes involving the silver ions that emerge at the surface from the conductive route underneath the porous material are increasingly significant to the total current.

The experiment indicates that the Ag-engendered faradaic ionic exchange is not expected to occur during the actual stimulation sessions, as the stimulating electrodes are subjected to waveforms that correspond to voltage sweep rates of the order of 10^4-10^5 V/s and in ranges that should be kept away from the water window limits.

[S1] Hadjinicolaou A E, Leung R T, Garrett D J *et al* 2012 Electrical stimulation of retinal ganglion cells with diamond and the development of an all diamond retinal prosthesis *Biomaterials* **33** 5812-5820

[S2] Norlin A, Pan J and Leygraf C 2005 Investigation of Electrochemical Behavior of Stimulation/Sensing Materials for Pacemaker Electrode Applications, I. Pt, Ti, and TiN Coated Electrodes *J. Elchem. Soc.* **152** J7-J15

5. Planar substrate probes—*Stimulation endurance tests*

Supplementary Table S2. Synthetic presentation of the stimulation endurance testing protocol and measurement results for the 5 electrodes (E1-E5) tested in vitro.

	E1	E2	E3	E4	E5
**Before stress test**					
Anodic activation	[0.2, 2] V	[0.2, 2] V	[0.2, 2] V	[0.2, 2] V	[0.2, 2] V
$\|Z\|_{EEI- 1 kHz}$ (Ω)	484	359	370	364	304
$\|Z\|_{EEI- 10 Hz}$ (Ω)	39516	30531	31138	31209	29680
Symm. activation	na	[-2, +2]V - 3 cy.	na	na	na
CS_C [mC·cm^{-2}]	2.87	83.01	3.46	3.10	3.25
**Stress test**					
$\|Z\|_{1 kHz-ini}$ (Ω)	1290	278	1130	1180	1040
Pulse activation	1…10 mA	1…10 mA	1…10 mA	1…10 mA	1…10 mA
$\|Z\|_{1 kHz-act}$ (Ω)	647	275	921	870	824
$V_{ave-act (10mA)}$ (V)	3.6	2.7	3.5	3.9	3.3
Stress test	10 mA (2 h)	10 mA (1 h)	10 mA (0.5 h)	7 mA (1 h)	4 mA (1 h)
$\|Z\|_{1 kHz-fin}$ (Ω)	256	275	381	462	497
$V_{ave-fin (10mA)}$ (V)	2.9	2.7	3.0	3.3	3.0
**After stress test**					
$\|Z\|_{EEI- 1 kHz}$ (Ω)	10	6	275	383	190
$\|Z\|_{EEI– 10 Hz}$ (Ω)	2316	470	25006	31466	14596
CS_C [mC·cm^{-2}]	16.34	71.12	3.67	3.14	4.25

To assess the behavior under realistic current-controlled stimulation pulses, an endurance test was conducted in vitro (PBS solution) on a planar configuration (Figure S9) comprising five freshly fabricated electrodes (E1-E5).

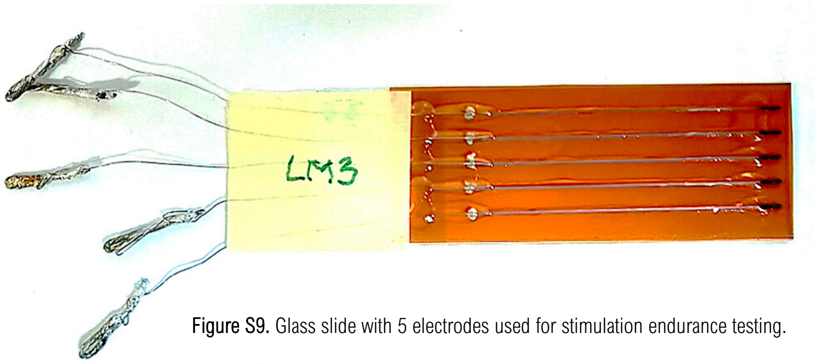

Figure S9. Glass slide with 5 electrodes used for stimulation endurance testing.

Supplementary Table S2 summarizes the testing protocol used, and the main results. In the pre-test and after-test phases, the electrodes were characterized by the EIS-fitted electrode–electrolyte impedance modulus $|Z|_{EEI}$ at 1 kHz and 10 Hz, and the CV-deduced specific cathodic charge storage CS_C. Also, the measured 1 kHz impedance $|Z|_{1 kHz}$, and the driving voltage half-ranges at 10 mA pulse amplitude $V_{ave(10mA)}$, were recorded before and after the stress procedure.

- Before the stress endurance test, all electrodes were anodically pre-activated; additionally, for one of the electrodes (E2), an additional, 3-cycle, symmetric-potential activation (between -2 and +2 V) was performed in the pre-test phase. All five carbonic contacts were analyzed by EIS, and CV (between [-1.4, +1.4] V, at 50 mV/s). The EIS spectra were fitted with the best equivalent circuit used throughout this report and the electrode–electrolyte interface impedance

($|Z|_{EEI}$) was calculated at 1 kHz and 10 Hz. The specific cathodic charge storage (CS_C) was also calculated and recorded at this pre-testing step.

- After these preliminary operations, the electrodes were kept in open air for 7 days.

- The actual endurance test was preceded by a pulse activation step for all electrodes, as it was observed that subjecting electrochemically activated contacts to pulsed current waveforms significantly reduces their 1 kHz measured impedance and the excursion of the driving voltage required to deliver a given stimulation pulse. The pulse activation step consisted in continuously increasing the current pulse amplitude from 1 to 10 mA in a 30 seconds interval. Initial and post-activation 1 kHz impedance measurements using the Guideline 5 system were performed ($|Z|_{1\ kHz\text{-}ini}$ and $|Z|_{1\ kHz\text{-}act}$, respectively). The test procedures used cathodic-leading, biphasic, pulsed current with 150 Hz frequency and 120 μs phase duration. To evaluate the effect of the stress level, the testing conditions were different between the electrodes. The mostly stressed electrode (E1) received 10 mA amplitude pulses that were maintained for 2 hours, which is by far exceeding the straining in an EM procedure, while the least stressed electrode (E5) was stimulated at a fair current amplitude (4 mA), though for a longer time (1 hour) than during a typical surgery. During the tests, no bubble formation was observed on either of the tested electrodes. The post-stress 1 kHz impedance was also measured ($|Z|_{1\ kHz\text{-}fin}$). Also measured by the Guideline 5 system were the driving voltage excursions at 10 mA pulse amplitude, before and after the stress tests ($V_{ave\text{-}act(10mA)}$, $V_{ave\text{-}fin(10mA)}$); these are listed in the table as directly reported by the system, i.e., as the mathematical averages of the absolute potential peaks (cathodic and anodic) reached in the pulse phases—that is, the driving voltage half-ranges.

- After the stress test, all electrodes were characterized again by CV and EIS as in the pre-testing stage.

- At the beginning and end of each step, the electrodes were washed with deionized water.

Figure S10 comparatively displays the CV scans before and after the pulsed current stress test.

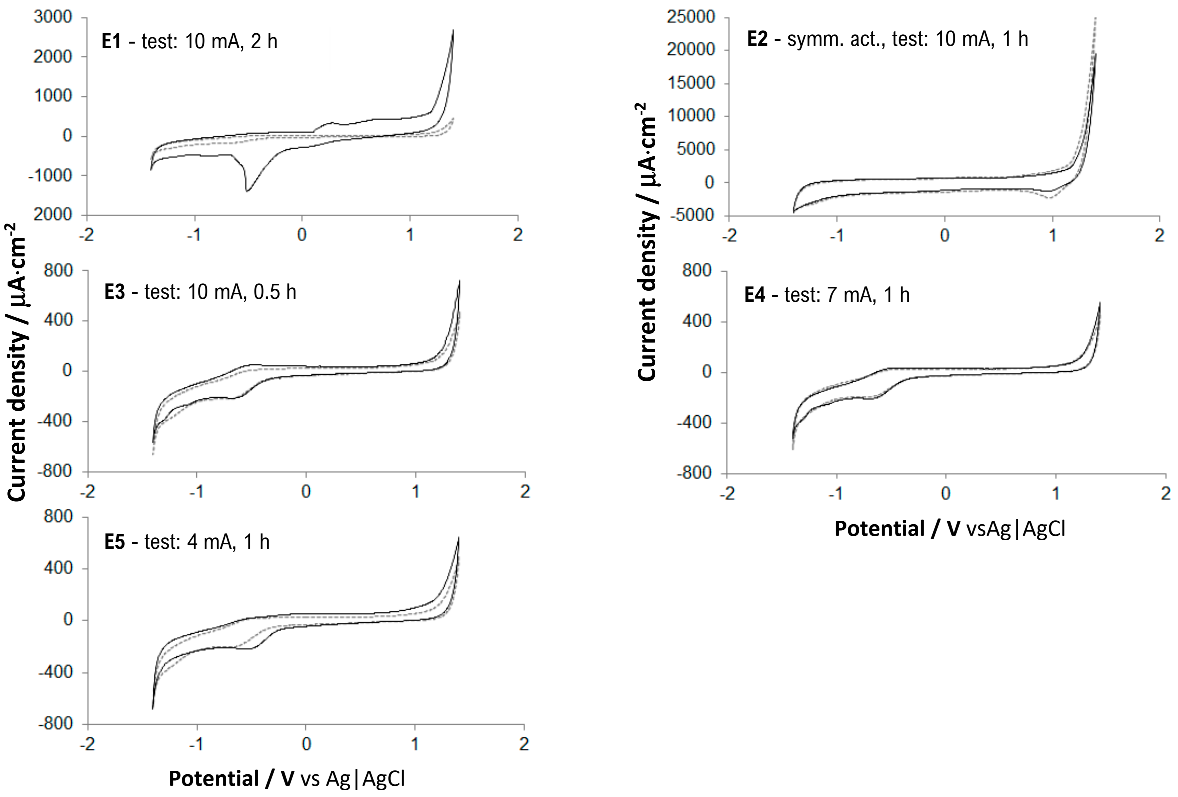

Figure S10. CV scans measured on each tested electrode before (*dashed gray traces*) and after (*continuous black traces*) the stress testing. Potential window: [-1.4, +1.4] V. Scan rate: 50 mV/s. GSA: 1 mm^2.

By cross-examining the synthetic data presented in Supplementary Table S2 and the scan plots, we notice in the first place that the only electrode that did not undergo any reduction in $|Z|_{1\ kHz}$ or $V_{ave(10mA)}$ throughout the test was the one which had been pre-activated with a [-2, +2] V symmetric waveform (i.e., E2). In contrast, all the other electrodes were progressively activated by the pulsed current waveforms.

Additionally, the $|Z|_{EEI}$ and the CS_C values before and after the pulsed current tests, corroborated with the CV sweeps in Figure S10, indicate that these changes majorly occurred in the high-frequency range; the exception being electrode E1, which received the most stressful current test. This observation evidences the efficiency of the cathodic–anodic electrochemical activation against the anodic-only one.

Furthermore, the ameliorations observed after the pulsed current sessions—in terms of the 1 kHz impedance and voltage excursions—combined with the results related to high-frequency, wide-range CV scans (illustrated by Figure S8), are motivating further investigations on the issue of optimizing the charge injection capacity specifically for the actually employed regime of pulsed current waveforms, vs. for the low-frequency voltage scans regime; i.e., it is expected that the chemo-structural modifications that occur in the graphitic material (immersed in neutral electrolytes) when exposed to high-frequency current biphasic pulses, versus low-frequency voltage scanning waveforms, present such differences that the parameters used for the electrochemical activation process must be chosen in correlation with the intended application of the electrode.

On the other hand, observing again the synthetic values from Supplementary Table S2, a reasonable predictive-confirmative quality of the slow-rate CS_C can be also noticed: when the

$|Z|_{1\ kHz}$ and $V_{ave(10mA)}$ values are checked horizontally (inter-electrode) and vertically (pre-test and post-test) in the table, a relatively fair correlation is found with the estimated inter-electrode ranking based on the corresponding pre-posttest CS_C values.

Regarding the comparative pre-posttest voltammogram profiles (Figure S10), we observe two outliers: for electrode E1, the high level of electrochemical stress applied resulted in a significant change of the CV profile, probably due to a combination of factors, such as overactivation under pulsed current and faradaic processes involving the silver ions of the underlying route; for E2, the initial additional activation in a symmetric voltage window seemed to shape a stable, hyperactivated carbonic surface. Note also in Supplementary Table S2, the drastic contrast of the $|Z|_{EEI}$ and CS_C final values for the E2 electrode against the other electrodes but also the accompanying water window decrease (Figure S10).

As exemplified in Figure S11, the stress tests did not affect the optically apparent surface morphology, or adherence, of the electrodes.

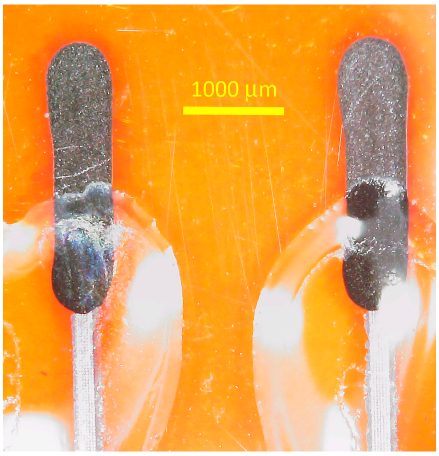

Figure S11. Optical image after current stress tests, channels E1 and E2. The epoxy protective layers are seen partially covering the carbonic contact.

6. Planar substrate probes—*In vitro recording tests*

In the planar electrode mockup, a preliminary recording test was conducted in vitro to evaluate the voltage pick-up performance in the low-frequency range, as is the case of LFP signals (few Hz, up to ~100 Hz). The simple model [S1] consisted in placing one planar, pulse-activated contact, and a reference wire in PBS solution in proximity with a pair of wires connected to the signal synthesizer to realize a floating voltage configuration. Sinusoidal waveforms of 30 Hz and 35 Hz, and 100 mVpp amplitude were generated through the synthesizer, and the signal was recorded with a professional system (see Materials and Methods section). The observed pick-up of the power line frequency was eliminated by applying a 1-40 Hz bandpass filter. As shown in Figure S12, this preliminary recording test indicates good quality, undistorted floating voltage pick-up.

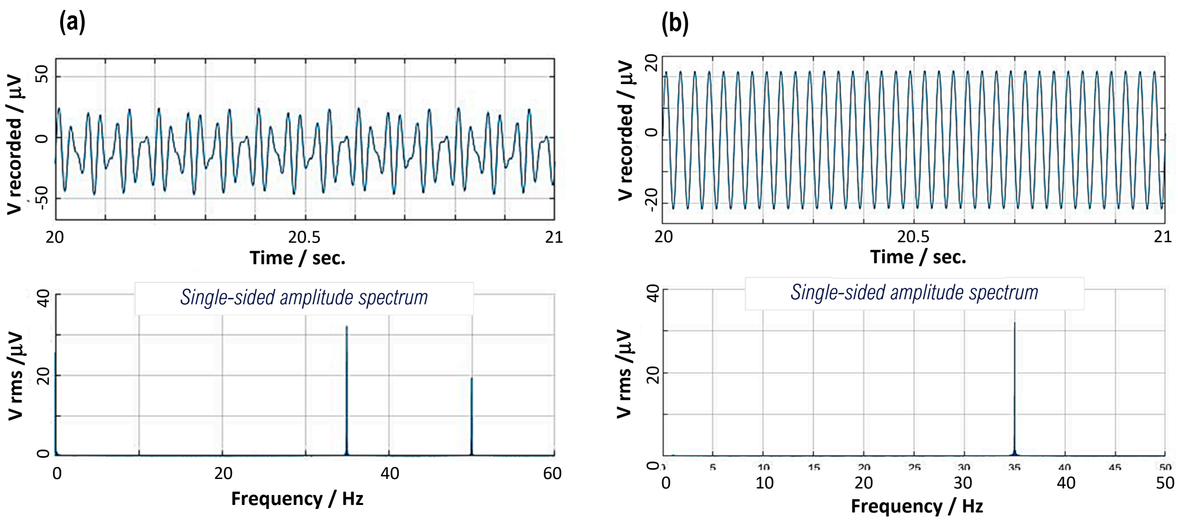

Figure S12. Test recording *in vitro* of a 35 Hz, 100 mVpp, harmonic voltage waveform generated as floating output to the recording chain. (a) Raw (unfiltered) signal pick-up; (b) bandpass filtered signal (1-40 Hz). Figures *top*: recorded signal. Figures *bottom*: spectral composition of the recording.

[S1] Rossi L, Foffani G, Marceglia S, Bracchi F, Barbieri S and Priori A 2007 An electronic device for artefact suppression in human local field potential recordings during deep brain stimulation *J Neural Eng* **4** 96-106

7. Cylindrical substrate probes—*Charge injection capacity (chronopotentiometry)*

The analyzed current amplitudes were chosen as 1, 3, 5, and 9 mA. A short analysis shows that applying 1 mA vs. 10 mA amplitude pulses over a 150 µs phase duration leads to 0.15 µC vs. 1.5 µC per phase, respectively; dividing these values to a 1 mm^2 GSA of the charge delivering contact, the corresponding delivered charge densities per phase read as 15 µC/cm^2 vs. 150 µC/cm^2 per phase, respectively. The usually cited tissue damaging threshold of 30 µC/cm^2 may represent a conservative safety limit, and additional factors will increase or decrease this limit, such as real (electrochemical) surface area, morphology, and geometry of the electrode, current density, pulse frequency, duration of the procedure, as well as the charge per phase.

Before the actual measurements, the electrodes were activated using increasing 1-10 mA pulse amplitudes for 30 seconds. For each chronopotentiometry measurement, trains of 10 current pulses were evaluated as sufficient for a stable voltage waveform.

8. In vivo experiments—*electrode preparation*

For the in vivo recording, stimulation, and 1 kHz impedance measurements tests, three freshly fabricated electrode probes were employed (550 µm outer diameter). Each probe was 10 cm long and consisted of two segmented electrode channels, fabricated to provide 180° oriented views.

Before insertions, each channel contact was activated in vitro (PBS solution) for about 30 seconds using cathodic-leading biphasic current pulses (120 µs phase duration, 150 Hz pulse frequency, no interphase gap) of increasing amplitude (1-10 mA). The activation was performed in a two-electrode setup by using the Guideline 5 clinical system, that was subsequently

employed for the in vivo testing. The system provided also the initial, and post-activation values of the total impedance at 1 kHz, and the excursions of the driving voltage, which were consistent with the previous three-electrode experiments, i.e., a decrease from 1-2 $k\Omega|_{1kHz}$ before the activation, to 6-900 $\Omega|_{1kHz}$ after the activation, while the driving voltage half-ranges decreased from 3-5 $V|_{10mA}$ to 2-3 $V|_{10mA}$.

9. Emulative experiment for the in vivo overpotential

Literature models for batteries and fuel cells

In [S1] an analytical approach is developed and applied, for the ion conduction mechanism at a discrete level in porous-tortuous path structures such as separator membranes and gel electrolytes; the approach is based on modeling the process of diffusion as a viscous flow, leading to a link between ion mobility and an equivalent microviscosity that accounts for morphology-restricted ion pathways. In [S2], the functional drawbacks of low diffusion rates of Li-ions through suboptimally designed porous-tortuous morphologies specific to battery electrode coatings are investigated, where the effective diffusion coefficient (and thereby, the mobility) is directly affected by both the low porosity, and high tortuosity, of the ion pathway. And in [S3], a model is developed to determine the influence of various parameters—such as porosity, tortuosity, pore diameter, and current density—on the anode diffusion polarization, which affects the efficiency of solid oxide fuel cells.

[S1] Saito Y 2021 Ion Transport in Solid Medium - Evaluation of Ionic Mobility for Design of Ion Transport Pathways in Separator and Gel Electrolyte *Membranes* **11** 277

[S2] Gao H, Wu Q, Hu Y, Zheng J P, Amine K and Chen Z 2018 Revealing the Rate-Limiting Li-Ion Diffusion Pathway in Ultrathick Electrodes for Li-Ion Batteries *J. Phys. Chem. Lett.* **9** 5100−5104

[S3] Zouhri K, Lee S-Y 2016 Exergy study on the effect of material parameters and operating conditions on the anode diffusion polarization of the SOFC *Int. J. Energy. Environ. Eng.* **7** 211–224

Literature models for the nervous tissue

Intensive data collection and processing are being conducted in the literature to model the structural complexity of the nervous tissue [S4,S5]—recently based on diffusion-weighted magnetic resonance imaging (water diffusion tensor images), to obtain tractographic reconstructions. Still, this research remains mainly focused on specific purposes, such as the mapping of the isotropic and anisotropic electrical conductivity for determining the volume of tissue activated by the electric field generated by DBS electrodes. Though, presently, the estimations for even the conductivity values—in isotropic and homogeneous approximation—have a broad dispersion in the literature [S5,S6].

[S4] Horn A *et al* 2019 Lead-DBS v2: Towards a comprehensive pipeline for deep brain stimulation imaging *NeuroImage* **184** 293–316

[S5] Baniasadi M, Proverbio D, Gonçalves J, Hertel F and Husch A 2020 FastField: An open-source toolbox for efficient approximation of deep brain stimulation electric fields NeuroImage **223** 117330

[S6] McCann H, Pisano G and Beltrachini L 2019 Variation in Reported Human Head Tissue Electrical Conductivity Values *Brain Topography* **32** 825–858

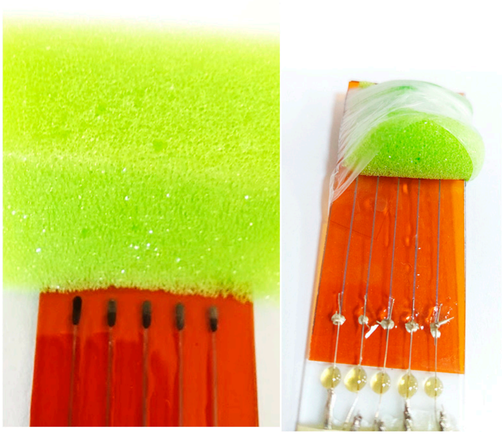

Figure S13. ***Left***: Carbonic electrodes in planar geometry, with the spongiform structure used for the experiment. ***Right***: Fastening of the sponge over the electrode area (medium tightening case).

Prior to the experiment, the sponge was thoroughly washed with isopropyl alcohol, then rinsed in deionized water, and finally sonicated in deionized water for 30 min., and well dried in nitrogen flow. Before the measurements, the ensemble was left in PBS for 30 min. to stabilize the saturation of the pores with fluid.

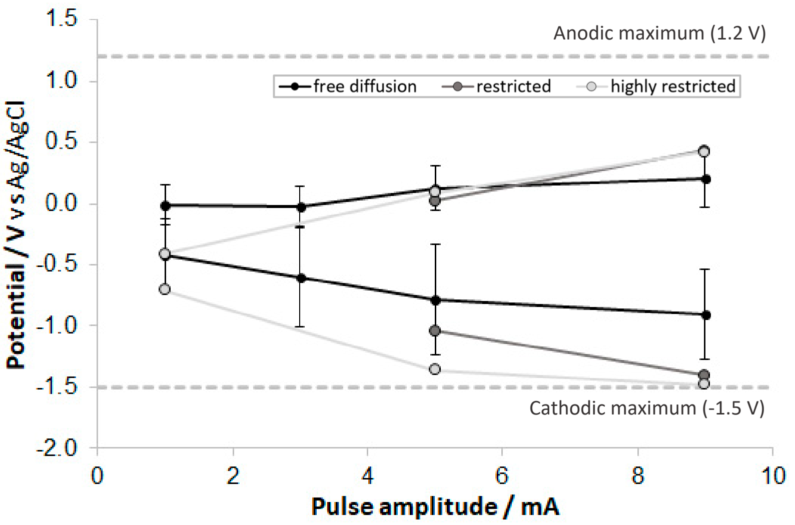

Figure S14. Comparative overlay (with the free-diffusion cumulative data presented in Figure 7e) of the V_{acc}-corrected polarization extremes (V_C+V_{acc}, V_A-V_{acc}) calculated from the pulse chronopotentiometry measurements (150 Hz biphasic pulses, 150 µs phase), after corrections for the access voltage, for the 1 mm^2 GSA carbon-contact electrode in PBS solution, for two levels of diffusive limitation. The error bars correspond to the standard deviation (n=5).
